# Structure of the Huntingtin F-actin complex reveals its role in cytoskeleton organization

**DOI:** 10.1126/sciadv.adw4124

**Published:** 2025-09-19

**Authors:** Rémi Carpentier, Jaesung Kim, Mariacristina Capizzi, Hyeongju Kim, Florian Fäßler, Jesse M. Hansen, Min Jeong Kim, Eric Denarier, Béatrice Blot, Marine Degennaro, Sophia Labou, Isabelle Arnal, Maria J. Marcaida, Matteo Dal Peraro, Doory Kim, Florian K. M. Schur, Ji-Joon Song, Sandrine Humbert

**Affiliations:** ^1^ Univ. Grenoble Alpes, Inserm, U1216, CEA, Grenoble Institute Neurosciences, 38000 Grenoble, France.; ^2^ Department of Biological Sciences, Korea Advanced Institute of Science and Technology (KAIST), Daejeon 34141, Korea.; ^3^ Sorbonne Université, Institut du Cerveau-Paris Brain Institute-ICM, Inserm, CNRS, AP-HP, Hôpital de la Pitié-Salpêtrière, Paris, France.; ^4^ Institute of Science and Technology Austria (ISTA), 3400 Klosterneuburg, Austria.; ^5^ Department of Chemistry, Research Institute for Convergence of Basic Science, Institute of Nano Science and Technology, Research Institute for Natural Sciences, Hanyang University, Seoul 04763, Korea.; ^6^ Institute of Bioengineering, School of Life Sciences, Ecole Polytechnique Fédérale de Lausanne (EPFL), 1015 Lausanne, Switzerland.

## Abstract

The Huntingtin protein (HTT), named for its role in Huntington’s disease, has been best understood as a scaffolding protein that promotes vesicle transport by molecular motors along microtubules. Here, we show that HTT also interacts with the actin cytoskeleton, and its loss of function disturbs the morphology and function of the axonal growth cone. We demonstrate that HTT organizes F-actin into bundles. Cryo–electron tomography (cryo-ET) and subtomogram averaging (STA) structural analyses reveal that HTT’s N-terminal HEAT and Bridge domains wrap around F-actin, while the C-terminal HEAT domain is displaced; furthermore, HTT dimerizes via the N-HEAT domain to bridge parallel actin filaments separated by ~20 nanometers. Our study provides the structural basis for understanding how HTT interacts with and organizes the actin cytoskeleton.

## Teaser

Huntington’s disease protein organizes F-actin into bundles.

## INTRODUCTION

Huntingtin (HTT) is a very large, well-conserved 348-kDa protein that was discovered for its role in Huntington’s disease (HD), where it contains an abnormal expansion of a polyglutamine tract in its N terminus (*1*). HTT is ubiquitously expressed and has hundreds of interactors involved in a range of functions, from transcription and RNA splicing to endocytosis, metabolism, cell division, and trafficking (*2*–*4*). This diversity reflects its central function as a scaffolding protein: for example, HTT facilitates intracellular transport by scaffolding vesicles on molecular motors as they travel along microtubule networks within neuronal axons (*5*). HTT relies on microtubules for many of its activities (*5*), but a few studies have reported that HTT loss of function or mutation also alters the behavior of another major cytoskeletal protein, namely, actin (*6*–*10*). HTT has been found to indirectly influence the actin cytoskeleton through interaction with the lysine methyltransferase SETD2 (HYPB) (*11*, *12*) and proteins of the Rho guanosine triphosphatase signaling pathway (*8*, *13*, *14*). Proteomic studies have also yielded interactions with several actin-binding proteins such as the actin monomer–binding protein profilin, the molecular motor myosin Va, the F-actin cross-linker α-actinin, and the huntingtin interacting proteins HIP1 and HIP1R (*8*, *15*–*19*). Consistent with this, HTT has been observed to colocalize with the actin cytoskeleton (*7*, *17*, *20*), and a short N-terminal fragment of HTT cosediments with F-actin (*21*). The molecular basis for HTT interaction with the actin cytoskeleton, however, as well as the purpose, remains elusive. To begin to answer these questions, we integrated functional studies using mouse cortical neurons lacking the murine *Htt* with biochemical and structural studies using full-length human recombinant HTT and various mutant constructs for cryo–electron tomography (cryo-ET) and subtomogram averaging (STA).

## RESULTS

### HTT associates with F-actin in the axonal growth cone

**Fig. 1.**
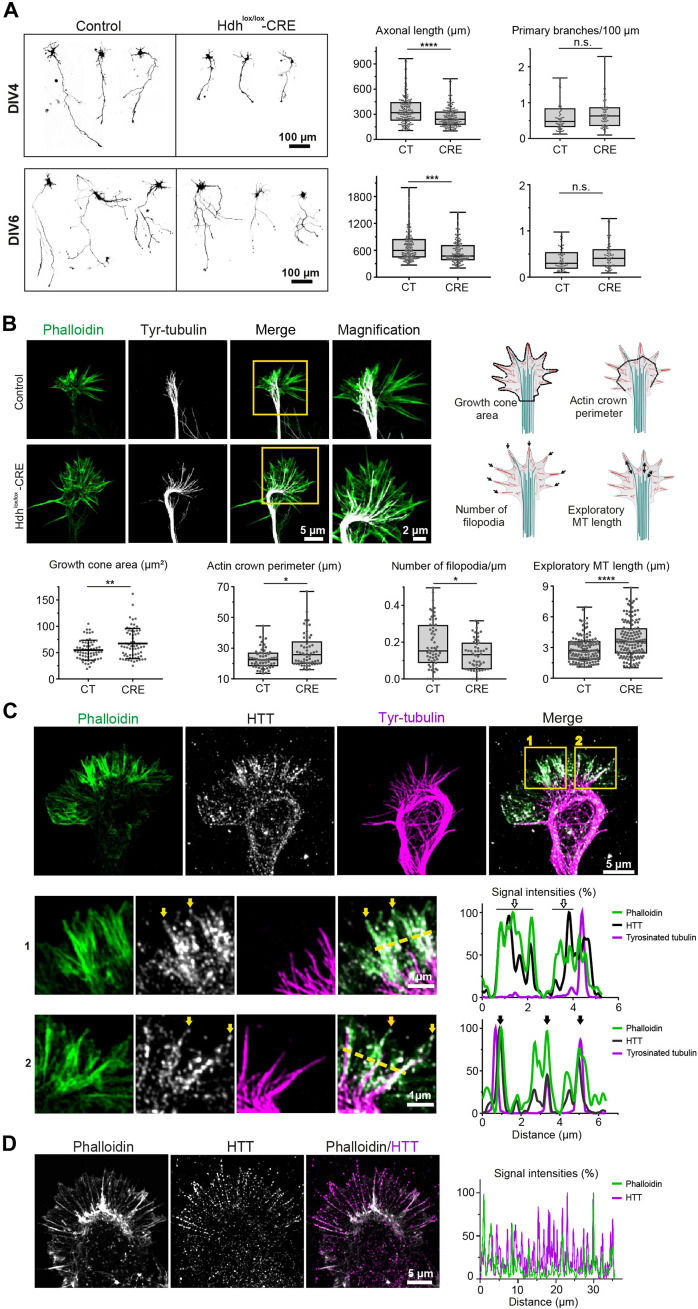
HTT is necessary for cytoskeletal organization in the axonal growth cone. (**A**) Left: Representative confocal images. Right: Length at DIV4, ****P < 0.0001, Mann-Whitney test, at least 20 axons per condition and experiment, three independent experiments (CT: 319.3 μm; CRE: 240.1 μm). Branching at DIV4, not significant (n.s.), Mann-Whitney test (CT: 0.48; CRE: 0.63). Length at DIV6, ***P < 0.001, Mann-Whitney test (CT: 599.10; CRE: 470.70). Branching at DIV6, n.s., Mann-Whitney test (CT: 0.30; CRE: 0.41). (**B**) Left: Representative airyscan confocal images of DIV4 growth cones. Yellow squares mark magnified areas. Right: Diagrams illustrate the parameters measured. Bottom: Growth cone area, **P < 0.01, unpaired *t* test (CT: 54.8 ± 18.9; CRE: 67.3 ± 28.6). Actin crown perimeter, *P < 0.05, Mann-Whitney test (CT: 22.5, CRE: 25.8); density of filopodia, *P* < 0.05, Mann-Whitney test (CT: 0.15; CRE: 0.13; at least 52 growth cones per condition); length of exploratory microtubules, Mann-Whitney test, ****P < 0.0001 (CT: 2.69 μm; CRE: 3.56 μm). At least 42 growth cones/genotype, six independent experiments. MT, microtubule. (**C**) Airyscan confocal image of a DIV4 growth cone. Areas 1 and 2 are magnified below in rows 1 and 2, respectively. HTT localizes with F-actin in filopodia in the absence of exploratory microtubules (row 1) and at the interface of F-actin and exploratory microtubules (row 2). Yellow arrows: HTT localized at the tip of filopodia; yellow dashed lines: axis along which the representative line scan analyses were performed. The right panels are representative line scan analyses (relative fluorescence intensity) of indicated immunostainings. Empty arrows (top graph): phalloidin/HTT colocalization; black arrows (bottom graph): triple colocalization of phalloidin, HTT, and tyrosinated tubulin. Data are shown as mean ± SD. (**D**) Left: Representative airyscan confocal acquisition of wild-type growth cone. Right: Line scan analysis of indicated immunostainings across the peripheral domain of the growth cone shown at left.

A recent study of the developmental origins of cortical defects in HD revealed that the axons in HD mice are shorter than in wild-type animals, with many axons not even crossing the corpus callosum (*22*). This growth defect corresponded to microtubule disorganization within the axonal growth cones, i.e., swellings at the axon tips that respond to attractive and repulsive chemical signals. Because axons grow through the interplay of actin filaments and microtubules (*23*), this finding suggested that the defect in microtubule-based vesicular transport observed in HD might involve disorganization of the actin cytoskeleton as well as microtubules. To explore this possibility, we depleted HTT in primary cortical neurons. HTT-depleted neurons showed significantly shorter axons at 4 days in vitro (DIV4) and DIV6, although the number of primary branches was similar (Fig. 1A). We labeled F-actin and microtubules and measured growth cone areas, actin crown perimeter, the number of filopodia, and the length of exploratory microtubules (Fig. 1B). Loss of HTT seemed to loosen the architecture of the growth cone: DIV4 HTT-depleted neurons displayed larger growth cone areas with fewer filopodia and a longer actin crown perimeter. The HTT-depleted neurons also had longer exploratory microtubules.

Immunofluorescence showed that HTT colocalized with both microtubules and F-actin throughout the growth cone but was particularly enriched with F-actin bundles in filopodia and along the interface between F-actin and exploratory microtubules invading filopodia (Fig. 1C). HTT seemed quite specific with regard to actin structures: It localized to F-actin bundles but not to the network of branched actin composing lamellipodia (Fig. 1D). We next verified that endogenous HTT associates with the cytoskeleton using detergent extraction to remove cytosolic proteins while allowing cytoskeletal proteins to remain attached (*24*). Whereas permeabilization decreased the mean fluorescence intensity of cytosolic and exogenously expressed fluorescent protein Venus within growth cones, the endogenous HTT remained colocalized with F-actin and microtubules (fig. S1).

### HTT binds directly to F-actin and organizes actin filaments into bundles

To determine whether HTT directly interacts with F-actin and microtubules, we purified recombinant human full-length wild-type HTT (21 glutamines) (*25*) and performed cosedimentation glycerol gradients with F-actin or microtubules (fig. S2A). Full-length HTT (FL-HTT) cosedimented with F-actin but not with microtubules in our in vitro condition. We determined the strength of the interaction by analyzing the fraction of FL-HTT found in the pellet after incubation with increasing concentrations of F-actin and high-speed cosedimentation (fig. S2, B and C). The calculated equilibrium dissociation constant (Kd_app_), which corresponds to the concentration of F-actin required to bind 50% of FL-HTT, was 236 ± 148 nM.

**Fig. 2.**
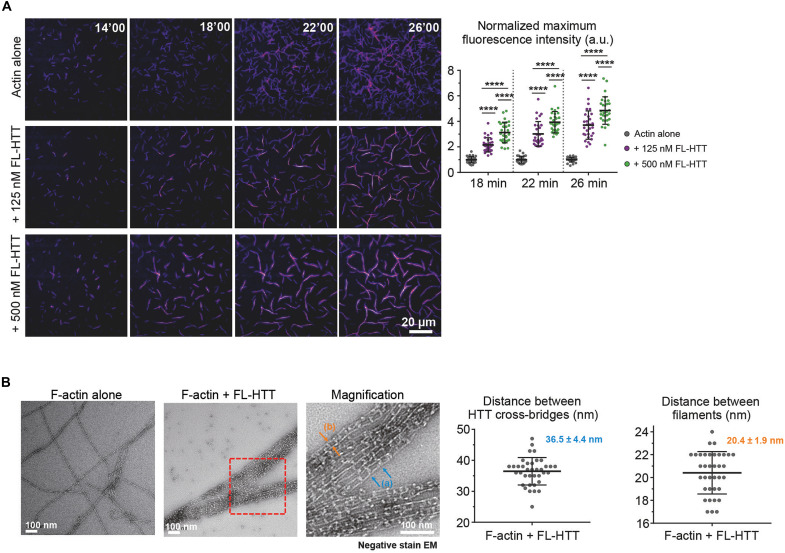
HTT binds and bundles actin filaments. (**A**) Left: Representative TIRF time-lapse images of ATTO565-actin polymerization with different FL-HTT concentrations, showing that the addition of HTT induces F-actin bundling. Right: The mean fluorescence intensity of the 10 brightest filaments/bundles at several time points in the absence of HTT or at different FL-HTT concentrations. One-way analysis of variance (ANOVA) followed by Tukey’s multiple comparisons post hoc test, ****P < 0.0001 (for actin alone: 1 at all times; for 125 nM: 2.155; 3.019; 3.712; for 500 nM: 3.124; 3.933; 4.850; *n* = 3 replicates with at least 10 filaments per condition per replicate). (**B**) Left: Representative electron micrographs of negative staining EM showing that the presence of FL-HTT causes F-actin filaments to organize into bundles. Zoom-in magnifications show F-actin bundles covered by FL-HTT molecules with a regular spacing between FL-HTT cross-bridges: Blue arrows (a) annotate the distance between two HTT cross-bridges, and orange arrows (b) annotate the cross-strand distance between two actin filaments within a bundle. Right: Measurements of the adjacent spacing between HTT cross-bridges (36.5 *±* 4.4 nm; *n* = 37) and the cross-strand distance between actin filaments within a bundle (20.4 *±* 1.9 nm; *n* = 36). Data are shown as mean ± SD. a.u., arbitrary units.

We next investigated whether HTT regulates F-actin organization. We used total internal reflection fluorescence (TIRF) microscopy to visualize the polymerization of fluorescently labelled ATTO565-actin in the presence of HTT (Fig. 2A and movie S1) and found that HTT induced bundling. Measuring the mean fluorescence intensity of the 10 brightest objects—single filaments in the control conditions and bundles at 125 and 500 nM FL-HTT for each time point—showed that bundles induced by 500 nM FL-HTT had a greater mean fluorescence intensity than those induced by 125 nM FL-HTT (Fig. 2A). Higher concentrations of FL-HTT thus induced a greater proportion of the F-actin network to organize into bundles.

We examined F-actin bundles induced by FL-HTT using negative stain transmission electron microscopy (nsTEM). F-actin alone showed a filamentous structure without any bundling, but in the presence of HTT, F-actin formed mesh-like bundles with HTT cross-bridges (Fig. 2B and fig. S3). The spacing between the HTT cross-bridges was 36.5 ± 4.4 nm, which corresponds to half a pitch of an F-actin helix (*26*). The mean distance between two adjacent actin filaments within a bundle was 20.4 ± 1.9 nm. Considering the height of FL-HTT is about 15 nm (150 Å) (*27*), it is likely that two HTT molecules dimerize to organize F-actin into bundles.

### Cryo-ET structure of the HTT F-actin complex

**Fig. 3.**
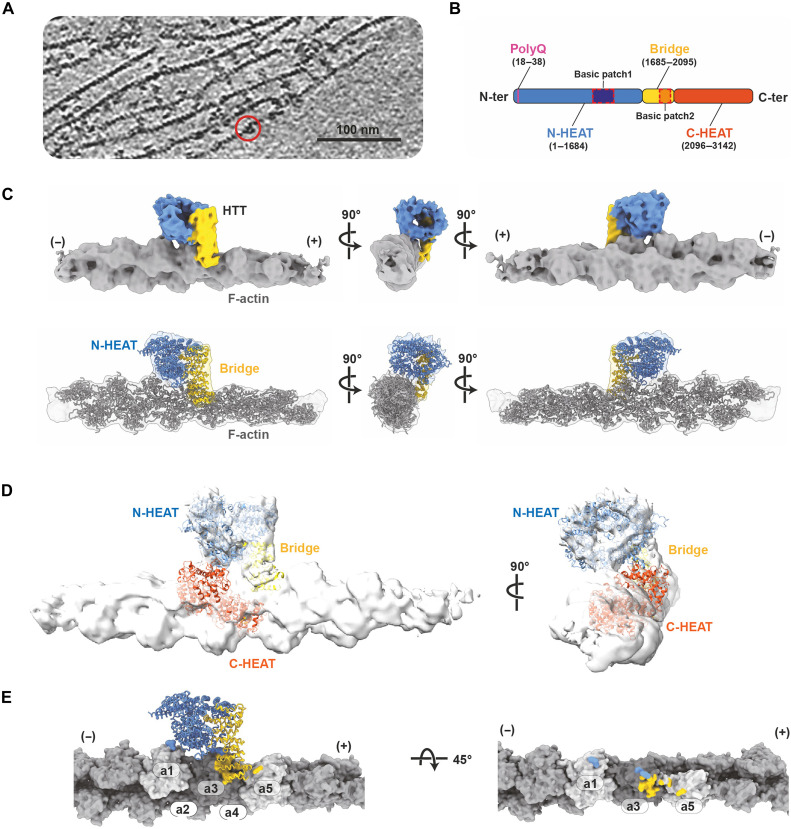
Cryo-ET of HTT F-actin complex. (**A**) A representative reconstructed tomogram of HTT bound to F-actin (the red circle marks an example bound HTT). Scale bar is annotated in the figure. (**B**) A diagram of the structural domains of HTT: N-HEAT (blue), Bridge (yellow), and C-HEAT (orange). A basic patch within the B-domain is indicated with an orange dotted box. Residue numbers are in parentheses. (**C**) Top: STA map of the HTT F-actin complex colored by domains. F-actin is in gray, and the N-HEAT and Bridge domains are in blue and yellow, respectively. The barbed end is indicated with (+) and the pointed end with (−). Bottom: The N-HEAT and Bride domains from the HTT (PDB ID: 6EZ8) are rigid-body fit into the cryo-ET map. (**D**) The position of C-HEAT shows a steric clash with F-actin. (**E**) HTT interacts with three F-actin subunits (a1, a3, and a5). Interaction surfaces of N-HEAT and Bridge domains are painted on F-actin in blue and yellow, respectively.

We next used cryo-ET followed by STA to determine how HTT binds and organizes F-actin (Fig. 3, figs. S4 and S5, and table S1). As seen by nsTEM, our cryo–electron tomograms displayed F-actin bundles, where individual F-actin filaments were connected via protein densities, which is presumably HTT. We also visualized HTT densities bound to the surface of actin filaments without contacting a second filament (Fig. 3A). Using STA, we first obtained a structure of the densities bound to a single actin filament at a global resolution of 10.1 Å (Fig. 3, B and C). This resolution allowed unambiguous fitting of F-actin into the EM density (Fig. 3C and fig. S6A). The remaining unoccupied density displayed a solenoid shape on the side of F-actin, which accommodated the N-HEAT domain of HTT [derived from the huntingtin-associated protein 40 (HAP40) complex, Protein Data Bank (PDB) 6EZ8] (Fig. 3C and movie S2) (*28*). The HTT bridge domain could be fitted into the map via a rigid body refinement with rotational movements of 10° and 25° at the end of the N-HEAT domain (fig. S6B). Notably, no density for the C-HEAT domain was observed. Comparing our generated F-actin/HTT model to the previously reported HTT-HAP40 complex, we found the C-HEAT domain to overlap with F-actin (Fig. 3D). Moreover, F-actin occupied a location topologically identical to that of HAP40 in the HTT-HAP40 structure (PDB 6EZ8) (fig. S6C). This strongly suggests that C-HEAT needs to be displaced to enable F-actin binding of HTT (fig. S6C) and that different binding partners of HTT may compete for the same binding site. In line with this, our glycerol gradient cosedimentation experiments showed that HTT-HAP40 cannot interact with F-actin (fig. S6D).

The cryo-ET structure revealed the molecular features of the HTT interaction with F-actin (Fig. 3E). HTT encompasses five actin filament subunits for its binding while directly interacting with three actin filament subunits. The Bridge domain binds between subdomains (SDs) 1 and 3 of actin subunit #3 (*29*) and SD2 of the next actin subunit toward the barbed end (actin #5). The N-HEAT domain is positioned toward SD2 of actin subunit #3 and SD1 of actin subunit #1. These data show that HTT binds to F-actin on conserved sites, as similar interfaces are regularly bound by other actin-binding proteins, including actin nucleators, cross-linking proteins, and motor proteins (fig. S7).

### HTT interacts with F-actin through its N-HEAT and Bridge domains

**Fig. 4.**
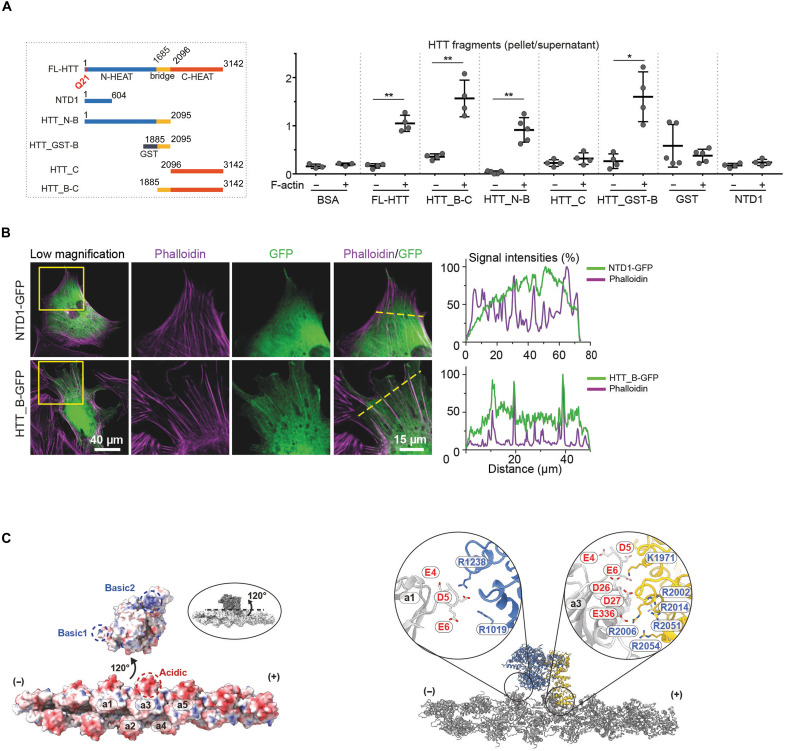
HTT Bridge domain is responsible for F-actin binding. (**A**) Left: Diagram of generated HTT fragments. N-HEAT, Bridge, and C-HEAT domains of HTT were colored in blue, yellow, and red, respectively. Right: The amount of HTT fragments found in the pellet relative to the supernatant, with or without F-actin. Paired *t* test, *P < 0.05 and **P < 0.01 (*n* ≥ 3). (**B**) Left: Representative confocal images of MEFs transfected with NTD1-GFP or HTT_B-GFP (green) and immunostained with phalloidin (magenta) to label F-actin. Yellow squares mark the area of higher magnification, and yellow dotted lines indicate the axis along which the representative line-scan analyses were performed. Right: Representative line-scan analyses (relative fluorescence intensity) of indicated immunostainings. (**C**) Left: Electrostatic surface representation of the interface between HTT and F-actin, visualized as if they were detached. The dashed circles indicate the interfaces which were in contact before being detached. Right: Detailed interaction between HTT basic1 and 2 patches with the acidic region of F-actin.

To further characterize the interaction between HTT and F-actin, we produced HTT fragments containing the N-HEAT and Bridge domains (HTT_N-B; 1 to 2068 amino acids), the Bridge and C-HEAT domains (HTT_B-C; 1885 to 3142 amino acids), the Bridge domain tagged with glutathione *S*-transferase (GST; HTT_GST-B: 1885 to 2095 amino acids), and the C-HEAT domain (HTT_C; 2096 to 3142 amino acids) (Fig. 4A). We also produced a partial fragment of the N-HEAT domain (NTD1, 1 to 604 amino acids) that was previously defined as N-terminal domain 1 using cross-linking mass spectrometry (*27*), as we were not able to generate the whole N-HEAT domain by itself. We assessed the ability of each of these fragments to bind F-actin by high-speed cosedimentation assays and glycerol gradient ultracentrifugation (Fig. 4A and fig. S8, A and B). In all cases, the HTT_N-B, HTT_B-C, and HTT_GST-B fragments cosedimented with F-actin as FL-HTT did. NTD1 and HTT_C did not appear to strongly bind F-actin. These data suggest that the Bridge domain is the major binding region for F-actin.

To determine whether the Bridge domain is sufficient to bind to F-actin in cells, we transfected mouse embryonic fibroblasts (MEFs) with constructs encoding the NTD1 [NTD1–green fluorescent protein (GFP)] and Bridge domain (HTT_B-GFP) tagged with GFP. Confocal microscopy revealed that NTD1-GFP diffused throughout the cytoplasm, whereas HTT_B-GFP adopted a filamentous pattern (Fig. 4B). Line scan analyses showed that the mean fluorescence intensity of NTD1-GFP was distributed throughout the cell without correlating with phalloidin, while HTT_B-GFP intensity peaks coincided with those for phalloidin (Fig. 4B). The Bridge domain therefore seems sufficient to mediate HTT binding to F-actin in cells.

### Basic patches in the Bridge and N-HEAT domains mediate F-actin binding 

In our cryo-ET model, HTT encompassed five actin filament subunits for its binding and directly interacted with three actin filament subunits. Two small contact sites of the N-HEAT domain are formed with actin subunits #1 and #3, while a more extensive interaction surface is formed between the Bridge domain and actin subunits #3 and #5 (Fig. 4C). Although the resolution of our cryo–electron microscopy (cryo-EM) density was too low to visualize individual side chains, our model strongly suggests that two basic clusters located in the N-HEAT and Bridge domains are involved in these interactions: Basic1 (Arg^1019^, Arg^1238^, and the backbone nitrogens of Ala^1077^ and Trp^1078^) in the N-HEAT and Basic2 (Lys^1971^, Arg^2002^, Arg^2006^, Arg^2014^, Arg^2051^, and Arg^2054^) in the Bridge (Fig. 4C). The Basic1 cluster potentially interacts with an acidic patch of actin #1 (Glu^4^, Asp^5^, and Glu^6^) and the Basic2 cluster with an acidic patch of actin #3 (Glu^4^, Asp^5^, Glu^6^, Asp^26^, Asp^27^, and Glu^336^). To examine the contribution of the basic residues in HTT to actin binding, we mutated Arg^1019^ and Arg^1238^ to Glu in Basic1 (Basic1mut) and all basic residues (Lys^1971^, Arg^2002^, Arg^2014^, Arg^2051^, and Arg^2054^) to Ser in Basic2 (Basic2mut). Gel filtration profiles and circular dichroism (CD) analysis confirmed that the mutations in the basic region did not affect the overall structure of HTT (fig. S9A). We then examined the ability of Basic1mut and Basic2mut to bind F-actin by cosedimentation upon high-speed ultracentrifugation (fig. S9B). The Basic1mut HTT was as competent as FL-HTT to bind F-actin, but the Basic2mut lost this ability, indicating that Basic2 within the Bridge domain is necessary for HTT/F-actin interaction.

### HTT dimerizes through its N-HEAT domain to bridge parallel actin filaments

**Fig. 5.**
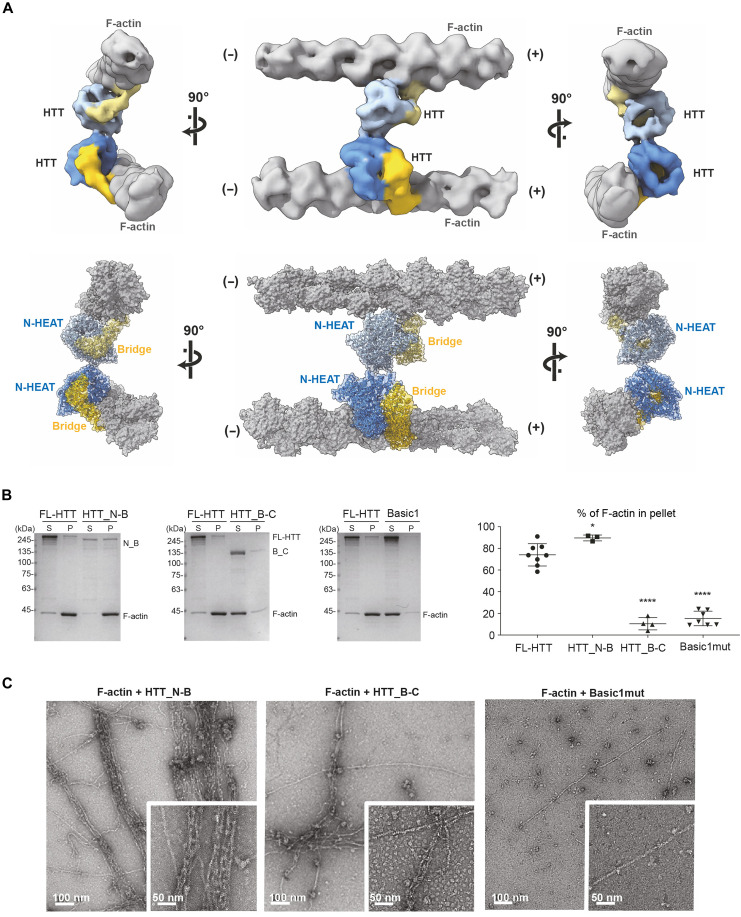
HTT bridges parallel actin filaments via dimerization of its N-HEAT domain. (**A**) Top: STA map of two F-actin filaments bundled in parallel orientation by a HTT dimer. F-actin is in gray; N-HEAT is in blue; the Bridge domains are in yellow. (−) and (+) indicate the actin pointed end and the barbed end, respectively. Bottom: Molecular model of the HTT dimer complexed with F-actin. HTT is shown in a ribbon diagram model overlaid with its semitransparent surface representation, and F-actin in a space-filling model. (**B**) Left: Representative SDS-PAGE gels with Coomassie blue staining from low-speed cosedimentation bundling assays of F-actin with FL-HTT, HTT_N-B, HTT_B-C, and Basic1mut. S, supernatant; P, pellet. Right: Quantifications of low-speed cosedimentation assays. One-way ANOVA followed by Dunnet’s post hoc test, ****P < 0.0001 and *P < 0.05 (*n* ≥ 3). (**C**) Representative electron micrographs with negative staining show that HTT_N-B, but not HTT_B-C or Basic1mut, organizes F-actin into bundles. Data are shown as mean ± SD.

We next sought to structurally determine how HTT mediates bundling of actin filaments by focusing on densities corresponding to F-actin/F-actin bridges in our cryo–electron tomograms (Fig. 3 and figs. S4 and S5E). We obtained a cryo-EM density map at 20.8 Å, which accommodated two HTT F-actin complexes in the EM density, without the fit requiring any further adjustment (Fig. 5A). These two HTT complexes formed a dimer across a small N-HEAT/N-HEAT interface, bundling F-actin filaments in a parallel arrangement with a spacing of ~200 Å (20 nm), consistent with our measurements using nsTEM (Fig. 2B). Despite the relatively low resolution of our reconstruction, the ridge and groove of the entire actin filament model fits only for parallel actin filaments. On the basis of this structure, it seems unlikely that HTT could bind F-actin with two opposite orientations, since even at our relatively low resolution, the HTT fit was good enough to assign unambiguous N-HEAT and bridge orientations with a parallel F-actin arrangement. Nevertheless, our dimer structure might represent only one of several possible conformations (i.e., the angle between the dimers might vary given the rather small interface we see). Further structural studies will be required to understand the rigidity of the bundling.

The C-HEAT domain was also not visible in the dimer structure, supporting the notion that it moves to accommodate HTT binding to F-actin. The limited 20.8-Å resolution of our reconstruction, due in part to structural variability of the HTT/HTT dimer, did not allow the amino acids responsible for the HTT_N/HTT_N interactions to be resolved. Given the small size of the dimer interface at the N-HEAT domains, it is possible that some of the disordered loops near the N-HEAT domains contribute to HTT dimerization. We used biochemical analyses to further examine the capacity of HTT domains to bundle F-actin (Fig. 5, B and C). Low-speed cosedimentation and nsTEM analysis show that HTT_N-B bundled F-actin, which indicates that the C-HEAT domain is dispensable for this function. Although the HTT_B-C still binds to F-actin (Fig. 4A), it did not form bundles (Fig. 5C). The N-HEAT domain thus appears to be required for bridging two actin-filaments. The Basic1mut, which retains F-actin binding ability (fig. S9B), could not bundle F-actin (Figs. 4C and 5B). This suggests that the interaction between the Basic1 region and F-actin is critical in F-actin bundling but not binding.

We also used stochastic optical reconstruction microscopy (STORM) to acquire super-resolution fluorescence images of F-actin with and without HTT (fig. S9C). FL-HTT and N_B-HEAT increased the width of actin objects, while those assembled in the presence of B_C-HEAT or Basic1mut have a width similar to that of F-actin alone. Although Basic1mut retains the ability to bind F-actin, the reason it fails to induce bundling remains unclear. It is possible that the interaction between the Basic1 region and F-actin triggers a change in conformation or spatial orientation in HTT that prevents actin bundling.

### The actin bundling activity of HTT is required for axonal growth

**Fig. 6.**
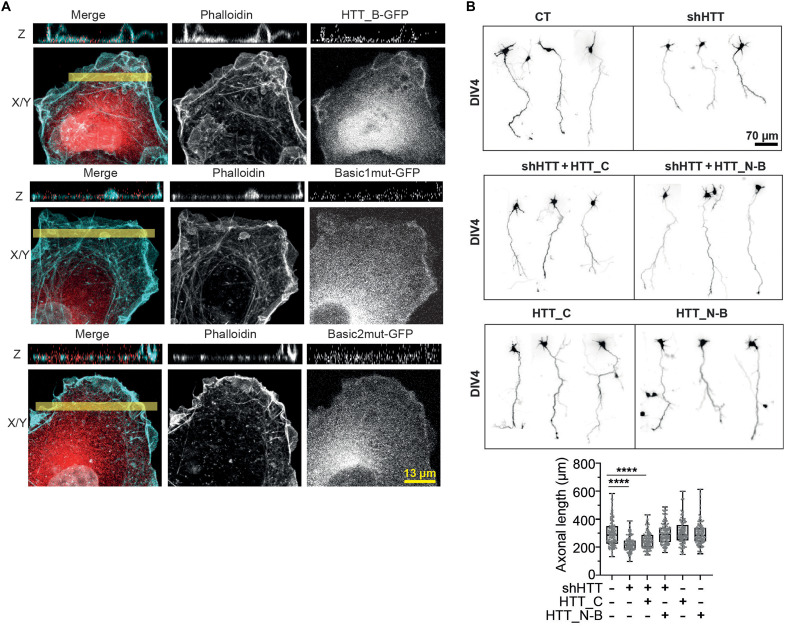
HTT regulates axonal growth through its actin bundling activity. (**A**) Representative confocal images of Cos7 cells transfected with HTT_B-GFP (top), Basic1mut-GFP (middle), and Basic2mut-GFP (bottom) and immunostained with phalloidin (cyan) to visualize F-actin. The yellow bar drawn in the *x*/*y* section bottom images denotes the position where a single *z* section (top image) is generated. (**B**) Top: Representative confocal images of neurons expressing the indicated constructs. Bottom: Length at DIV4, ****P < 0.0001, one-way ANOVA with Dunn’s multiple comparisons followed by Kruskal-Wallis test. (Mean of difference between control and shHTT, 219.2; between control and shHTT + HTT_C domain, 122.6; between control and shHTT + HTT_NB domain, −23.78; between control and HTT_C domain, −35.36; between control and HTT_NB domain, −13.84; two independent experiments, at least 50 axons per condition and experiment.)

To evaluate the physiological relevance of our biochemical and structural findings, we examined the effect of disrupting HTT F-actin binding interface using Basic1mut and Basic2mut. Because the large size of the HTT constructs precludes expressing them in neurons, we instead used COS-7 cells to assess their subcellular distribution relative to actin. As expected, the HTT_B-GFP localized in a filamentous pattern consistent with F-actin structures (Fig. 6). Similarly, Basic1mut-GFP retained this filamentous localization, but Basic2mut-GFP did not (Fig. 6A), indicating a disrupted interaction.

We then took advantage of two HTT fragments: HTT_N-B (1 to 2068 amino acids), which serves as a binding and bundling domain, and HTT_C (2095 to 3142 amino acids), which is deficient in actin binding. Expression of HTT_N-B, but not HTT_C, was sufficient to rescue axonal defects in HTT-depleted cortical neurons (Fig. 6B). We also investigated whether expression of HTT_N-B and HTT_C fragments in neurons with endogenous HTT would influence axonal growth. Neither fragment had a measurable effect under these conditions, showing that their impact is not dominant over the endogenous wild-type protein.

Given our previous observation of axonal growth defects in HD (*22*), we next investigated whether the abnormal polyglutamine expansion in mutant HTT may affect the protein’s ability to bind and bundle F-actin. We generated FL-HTT carrying a 78 polyglutamine stretch (Q78-HTT). Under our in vitro conditions, we did not detect a significant difference between Q21-HTT and Q78-HTT in their ability to bind and bundle F-actin (fig. S10).

## DISCUSSION

Here, we provide structural, biochemical, and cell biological evidence establishing HTT as an actin-binding protein that organizes F-actin into bundles of specific geometry. By cross-linking actin filaments, HTT may provide mechanical support and facilitate dynamic structures, which are critical for axonal growth cone motility. Beyond neuronal development, actin bundles play a key role in processes such as cytokinesis, cell adhesion, and mechanotransduction signaling, highlighting the broader implications of HTT/F-actin bundles in cellular division, cell motility, and tissue homeostasis.

HTT occupies the same position on F-actin as members of the myosin protein family, the actin filament nucleator Arp2/3 complex, or the actin bundling protein fascin. This suggests that HTT and other actin-binding proteins might compete for the same conserved F-actin binding site (fig. S7). It is tempting to speculate that the HTT/F-actin complex could define a specialized set of F-actin networks, e.g., by preferentially localizing to parallel bundled F-actin architectures over bundled filaments of mixed polarity such as contractile stress fibers enriched in myosin or Arp2/3-branched F-actin networks in lamellipodia. In support of this notion, the distance between HTT-bundled filaments is substantially larger than what is observed between parallel bundles induced by fascin or plastin (~12- to 16- nm interfilament distance) (fig. S11), two proteins that localize to filopodia (*30*–*33*). Moreover, fascin-mediated bundles in neuronal growth cone filopodia visualized by cryo-ET display an F-actin spacing that appears incompatible with HTT localization (*34*), and the relative orientations between actin filaments within HTT, fascin, and plastin-mediated bundles differ (fig. S11). HTT-bundled filaments are separated by ~20 nm (fig. S11), similar to what has been reported for actin filament cross-linkers such as α-actinin (*35*). Our observation aligns with a previous study that showed that HTT and α-actinin might interact and can both colocalize to stress fibers in human fibroblasts (*17*). Our data, however, can neither confirm nor exclude HTT localization to stress fiber–like actin assemblies, and future work will need to better characterize the exact localization of HTT in cellular actin networks.

Our structural studies show HTT forming into a multimer, with its N-HEAT domain mediating the dimer interface between two parallel F-actin filaments. The dimer interface is comparatively small, suggesting that other parts of HTT such as disordered loops and other region may be also involved in the dimerization. In line with the rather small dimer interface, the regularity of filament architecture was rather low, further exemplified by the low resolution we were able to obtain for our HTT dimer structure. This suggests the organization of HTT-mediated F-actin bundles to be pliable. Accordingly, our dimer structure might represent only one of many possible conformations that two HTT molecules might adopt in relation to each other to bridge actin filaments. Although our in vitro EM data suggest filament bundles to be formed over longer length scales, we do not have evidence to confirm this long-range bundling to be happening in cells. Studies using cryo-ET to describe the cytoskeletal architecture of neuronal growth cones have not reported the presence of HTT-like bundles (*36*), suggesting that such bundles may be localized to specific cellular subregions or occur at specific developmental stages.

It is also possible that the local enrichment of HTT in these positions fulfills other functions, since it is a scaffolding protein that could present proteins to other molecules. Considering the fact that the structure of HTT was originally solved in complex with HAP40 (*28*), which the current study shows occupies the same site as F-actin, HTT cannot bind to F-actin and HAP40 simultaneously. In addition, the C-HEAT domain is displaced upon F-actin binding but not in the HAP40 complex. It is therefore plausible that the exposed C-HEAT in the F-actin context may be primed for interactions with other protein partners yet to be identified.

The defects in axonal growth and in cytoskeletal organization observed in HD (*22*) suggest possible roles of HTT and F-actin interaction in HD pathology. Since the wild-type (Q21) and polyglutamine-expanded (Q78) HTT bind and bundle F-actin similarly in our in vitro experiments, and previous work showed that loss of HTT disrupted the axonal growth (*10*, *22*), it is likely that the haploinsufficiency of wild-type HTT function contributes to axonal growth defects in HD, in addition to the mutant form of HTT interfering with actin regulators in a specific spatiotemporal manner. We envision that the role of HTT in actin binding may be involved in a wide range of other biological process such as embryonic development (*37*–*39*) and muscle function (*40*).

## MATERIALS AND METHODS

### Purification of FL-HTT and HTT fragments

The expression and purification of FL-HTT have been described (*25*). We cloned the HTT fragments, including HTT_N-B, HTT_B-C, HTT_C, and NTD1, with a modified pFastBac1 vector (Thermo Fisher Scientific) containing N-terminal FLAG tag, 6 x His tag, and a Tobacco Etch Virus (TEV) protease cleavage site, and expressed them in Sf9 cells with Baculovirus. The purification method for HTT_N-B, HTT_B-C, and HTT_C was identical to the method for FL-HTT, but the purification of NTD1 was distinct from the other three fragments. From harvesting the expressed cells to purifying them, the NTD1 must be contained within a high salt buffer condition [500 mM NaCl, 5% glycerol, and 50 mM tris-HCl (pH 8.0)]. NTD1 was purified by affinity chromatography with Nickel-Nitrilotriacetic Acid (Ni-NTA) agarose (QIAGEN), followed by cleavage of N-terminal tags with TEV protease. After recapturing the cleaved tags with Ni-NTA agarose, the sample was purified with size exclusion chromatography using HiLoad Superdex 200 26/600 (Cytiva) within 500 mM NaCl and 50 mM tris-HCl (pH 8.0). The HTT B-domain cloned into a pGEX-4 T-1 vector was expressed with *Escherichia coli* BL21 (DE3) (Invitrogen) at 18°C in the presence of 0.5 mM isopropyl-β-d-thiogalactopyranoside for 18 hours. The incubated cells were harvested with centrifugation (4600*g* for 20 min) and resuspended in buffer containing 100 mM NaCl, 50 mM tris-HCl (pH 8.0), and 5% glycerol. The suspended cells were lysed with sonication, and cell lysates were cleared by centrifugation at 27,000*g* for 1 hour. The GST-tagged Bridge domain was purified by affinity chromatography with glutathione–Sepharose 4B resins (Cytiva) followed by size exclusion chromatography with Superose 6 10/300 (Cytiva) in the running buffer containing 150 mM NaCl and 20 mM Hepes (pH 7.5). The FL-HTT complex with HAP40 were purified as described previously (*25*).

### Cloning of strategy viral constructs HTT_C domain and HTT_N-B domain

The coding sequences of HTT_N-B and HTT_C domains were amplified by polymerase chain reaction (PCR) from the donor plasmid using primers with 15- to 20 bp overlaps complementary to the target viral vector (Trip-EF1short). The vector backbone was linearized by PCR (or restriction digestion) and assembled with the insert using Gibson Assembly Master Mix (New England Biolabs), following the manufacturer’s instructions. Correct assembly was confirmed by colony PCR and Sanger sequencing.

### Circular Dichroism (CD) analysis of FL-HTT and basic mutants

CD analysis was conducted on purified recombinant HTT proteins (FL-HTT, Basic1mut, and Basic2mut) at a concentration of 0.5 mg/ml in phosphate-buffered saline (PBS). Samples were placed in a 0.5-mm pathlength rectangular cell (JASCO Inc.) and measured using a JASCO J-815 spectropolarimeter. Spectra were collected from 185 to 285 nm with a wavelength pitch of 0.5 nm and a speed of 50 nm/min at room temperature (RT). Each sample was measured three times each to obtain the average spectrum. CD spectrums were smoothened using Spectra manager program (JASCO Inc.).

### F-actin preparation

Purified actin protein from rabbit skeletal muscle (Cytoskeleton Inc.) was depolymerized at 24 μM in actin depolymerization buffer containing 5 mM tris-HCl (pH 8.0), 0.2 mM CaCl_2_, 0.2 mM adenosine triphosphate (ATP), and 0.5 mM dithiothreitol (DTT) for 60 min on ice. After centrifuging at 17,000*g* for 15 min for clarification, the depolymerized actin was repolymerized to get filamentous actin (F-actin) with polymerization buffer containing 50 mM KCl, 20 mM imidazole (pH 7.0), 2 mM MgCl_2_, 1 mM ATP, and 1 mM EGTA for 45 min at RT.

### Cryo–electron tomography

#### Sample preparation

The HTT/F-actin sample for tomography was prepared by low-speed cosedimentation. First, polymerized F-actin and purified recombinant HTT were incubated in a 2:1 molar ratio within Brinkley Renaturing Buffer 80 (BRB80) buffer [80 mM Na-Pipes, 1 mM EGTA, 1 mM MgCl_2_, and 1 mM DTT (pH 6.7)] for 2 hours at 4°C. Then, the reaction was spun down at low-speed (14,000*g*) for 15 min, and the supernatant containing unbound HTT was discarded. The pellet surface was briefly washed with BRB80 buffer and resuspended with the same buffer.

Next, the purified bundles were stabilized via a cross-linking reaction. The resuspended sample was incubated with glutaraldehyde (0.2%, v/v) for 30 min at RT to stabilize the complex as used in the GraFix method (*41*), and the reaction was quenched by adding tris-HCl (pH 8.0) to a final concentration of 10 mM. For further purification of the bundle, the cross-linked sample was centrifuged and resuspended again as above. Before vitrification, prepared bovine serum albumin (BSA)–conjugated gold nanoparticles (*42*) were added to the sample. Next, 5 μl of the sample was applied to a nonglow discharged Quantifoil R 2/2 200 copper mesh grid and blotted under the following conditions (blotting force, −5; blotting time, 3 s; and waiting time, 3 s) using a Vitrobot Mark IV (Thermo Fisher Scientific). The blotted grid was frozen immediately in ethane slush and stored in liquid nitrogen until imaging.

#### Cryo-ET data acquisition

Tilt-series were acquired under cryogenic conditions on a Thermo Fisher Scientific Krios G3i transmission electron microscopy equipped with a BioQuantum postcolumn energy filter and a K3 camera (Gatan), using SerialEM v4.0.5. (*43*). Acquisition of low- and medium-magnification maps was performed to define areas of interest for subsequent high-resolution tomography data acquisition. Gain reference images were collected before data acquisition. DigitalMicrograph as integrated into the Gatan Microscopy Suite v3.3 (Gatan) and SerialEM were used for filter and microscope tuning. Tilt-series were acquired with a 50-μm C2 aperture, a 100-μm objective aperture, a filter slit width of 20 eV, and using a dose-symmetric tilt-scheme (*44*) ranging from −60° to 60° with a 2° increment. The nominal defocus range was set to −2 to −6 μm and the nominal magnification to ×64,000, resulting in a pixel size of 1.381 Å. Tilt-images were acquired as 5760 × 4092 pixel movies of eight frames. The dose per tilt was set to be 2.79 e/Å^2^, resulting in a calculated cumulative dose of 170 e/Å^2^ per series. Sixty tilt-series were acquired on one grid during one session. For data acquisition settings, see table S1.

#### Cryo-ET data processing

Gain correction, frame alignment, and defocus estimation of individual tilts were performed in Warp v1.0.9 nightly build 2020-11-04 (*45*). Before exporting tilt-series as mrc stacks, poor quality tilt-images caused, for example, by grid bars blocking the beam at high tilt-angles, were removed. Tilt-series alignment was done using gold fiducials (whenever possible) or patch tracking (when too few fiducial markers were available) within the IMOD software package v4.11 (*46*). Eighteen tilt-series were discarded because of bad alignment statistics, resulting in 42 tilt-series being used moving forward. Tilt-series alignment parameters as determined in IMOD were imported into Warp. The metrics necessary for dose-dependent low-pass filtering were calculated during this step. Defocus parameters were then again determined for the whole tilt-series. The 8× binned (11.048 Å/pix) tomograms were exported for template matching in the Dynamo package v1.1.333 (*47*). For undecorated actin filaments, a template was generated from a model of aged, nucleotide-bound, and phalloidin-stabilized F-actin [PDB 6T20 from (*48*)] using the molmap function in ChimeraX v1.3. The box of the resulting map was extended using RELION v3.0.8 (*49*, *50*) to be cube-shaped.

For template matching of HTT monomers bound to F-actin and HTT dimers bundling two actin filaments, positions (without angular information) of 241 and 532 particles, respectively, were picked manually within IMOD, on 8×-binned (11.048 Å/pix) tomograms treated by IsoNet v0.1. Particles (40 pix side length, 11.048 Å/pix) were then extracted from no–IsoNet-treated tomograms, aligned, and averaged in Dynamo to produce the respective references (*47*). For cross-correlation calculation during template matching, a cylindrical mask was applied to the undecorated actin filament to contain only F-actin. Spherical masks covering the HTT densities and neighboring actin moieties were used for HTT monomers and dimers. Angular scanning around all three Euler angles was performed for a full 360° with a sampling step of 10°. False-positive cross-correlation peaks from predetermined areas containing gray value outliers due to suboptimal correction for dead pixels in the tilt-series were automatically removed. Subsequently, up to 2000 undecorated F-actin stretches, up to 2000 HTT monomer, and up to 600 HTT dimer positions with the highest cross-correlation values and a minimum cross-correlation value of 0.05 per tomogram were selected for further processing, resulting in a total of 79,732, 84,000 and 24,228 particles, respectively.

For the HTT monomer dataset, an initial multireference alignment using four different classes was performed in Dynamo, reducing the particle count to 35,679 with three good classes. Dynamo2m scripts v0.2.2 (*51*) were used to transform particle coordinates from Dynamo to RELION format. Subtomograms (80 pix side length, 5.524 Å/pix) were exported using Warp. Three-dimensional (3D) classification in RELION was used to remove junk particles, reducing the number of particles to 59,680 for undecorated F-actin, 22,657 for HTT monomers, and 2854 for HTT dimers. These datasets were then subjected to RELION 3D auto-refine using averages determined from the current particle orientations (low-pass filtered to 40 Å) as reference. Particles were automatically distributed into even and odd subsets during the RELION workflow. Refinement for the HTT dimers was stopped after this step and resulted at a final resolution of 20 Å.

Undecorated F-actin and HTT monomer particles at the updated positions were extracted in bin2 (160 pix side length, 2.762 Å/pix) using Warp. 3D refinement in Relion resulted in maps at 9 and 10.5 Å for F-actin and the HTT monomer, respectively. Subsequently, multiparticle refinement was performed in M v1.0.9 nightly build 2020-11-04 (*11*), simultaneously considering F-actin and HTT monomer particles. Tilt-series were refined using image warp with a 9 by 6 grid and volume warp with a 4 by 6 by 2 by 10 grid, as well as tilt-angle optimization. Particle poses were refined for one temporal sampling point. Four iterations of refinement considering 80% of the available resolution were performed. Resolution at the 0.143 Fourier Shell Correlation (FSC) criterion was calculated with RELION postprocess using the halfmaps from RELION.

#### Model building

The molecular models of HTT F-actin complex was generated using HTT from PDB 6EZ8 and actin molecules from PDB 7NEP. Thirteen F-actin subunits and N-HEAT and Bridge domains were placed and refined with rigid body refinement using PHENIX (*52*).

### Data visualization

Figures 3 to 5 were prepared using ChimeraX version 1.6.1 (*53*). Lighting was set to “soft” graphics preset with silhouettes at default thickness. HTT domains are colored with ChimeraX standard color palette: N-HEAT (cornflower blue), Bridge (gold), and C-HEAT (red); actin is colored gray. The HTT-actin dimer (Fig. 5) was additionally colored with blue and yellow for the N-HEAT and bridge domains, respectively. Molecular models were displayed with ribbon width of 2.2 and thickness of 1. Molecular surface representations were visualized in ChimeraX with probeRadius 5, resolution 7, and gridSpacing 0.5.

### Cosedimentation of purified HTT with F-actin

For F-actin binding assay, 3 μM F-actin was incubated with 500 nM purified HTT proteins in a final volume of 20 μl of (actin polymerization, AP) buffer containing 20 mM imidazole, 100 mM KCl, 2 mM MgCl_2_, 0.5 mM ATP (Sigma-Aldrich), and 1 mM EGTA (pH 7.0) for 30 min at RT and then endured high-speed centrifugation using the TLA-100.3 rotor (Beckman Coulter) at 100,000*g* for 20 min at 23°C. The supernatant was collected, the pellet was resuspended and collected, and both fractions were then loaded onto 8% SDS–polyacrylamide gel electrophoresis (SDS-PAGE) for analysis after staining with Coomassie blue. The quantity of HTT found in the pellet was measured relative to the quantity of HTT found in the supernatant. To determine the apparent equilibrium dissociation constant *K*d_app_, a constant quantity of 500 nM FL-HTT was incubated with increasing concentrations of F-actin for 30 min at RT and centrifuged at high speed. Then, the supernatant and pellet were collected and loaded onto 8% SDS-PAGE for analysis after staining with Coomassie blue. The quantity of FL-HTT found in the pellet and supernatant was expressed as percentage of total FL-HTT. The fit of the binding curve was made as described for an intermediate regime accounting for ligand depletion and incomplete saturation (*54*).

### Glycerol gradient comigration assay with purified HTT

For comigration assay with glycerol gradient, FL-HTT or HTT fragments were incubated with F-actin in a 1:6 molar ratio, and HTT-HAP40 complex was incubated with 1:1 molar ratio for 2 hours at 4°C. Samples were submitted to separation on a continuous glycerol gradient (30 to 70%) in BRB80 buffer at 50,000 rpm for 2 hours at 4°C with a SW55Ti rotor (Beckman & Coulter). After ultracentrifugation, samples were fractionized to 200 μl and analyzed with SDS-PAGE, followed by Coomassie blue staining.

### Transmission electron microscopy of HTT-actin complex

The HTT-actin complex for nsTEM was prepared by incubating 3 μM F-actin in the presence of 500 nM HTT protein within BRB80 buffer for 1 hour at 4°C. F-actin and HTT complexes were purified from free HTT and actin by low-speed centrifugation (14,000*g*) for 15 min. Supernatants were discarded, and pellets were resuspended with 20 μl of BRB80 buffer. For negative staining, 5 μl of resuspension was loaded onto freshly glow-discharged 400-mesh copper grid (Graticules Optics) and incubated for 1 min. After incubation, excessive liquid was blotted using filter paper (Whatman no. 4) and sequentially washed twice with distilled water and floated on 2% uranyl acetate in water for 1 min and then air dried at air after blotting. Grids were imaged with a Tecnai F20 transmission electron microscope operating at 200 kV. Images were acquired with a charge-coupled device camera (Gatan) at magnification ranging from ×19,500 to ×71,000. Acquisitions were performed at the KARA (KAIST Analysis Center for Research Advancement).

### TIRF microscopy

Chambers were prepared from glass slides and functionalized with silane–polyethylene glycol (PEG; Creative PEGwork) as described previously. Before actin polymerization experiments, chambers were incubated with a solution containing 1.5-kDa PLL-g-PEG (0.1 mg/ml; Jenkem) in 10 mM Hepes (pH 7.4) for 2 min at RT and washed with AP buffer [10 mM Hepes (pH 7.4), 50 mM KCl, 5 mM MgCl_2_, and 1 mM EGTA] containing 1% BSA. Polymerization of 1.5 μM actin mix containing 12% ATTO565-labeled G-actin and 88% unlabeled actin (Hypermol) was monitored in the presence of 125 nM or 500 nM FL-HTT in TIRF buffer (AP buffer containing 4 mM DTT, 0.1% BSA, 1 mg/ml glucose, 82 μg/ml catalase (Sigma-Aldrich), 0.58 mg/ml glucose oxidase (Sigma-Aldrich), 0.25% methylcellulose (Sigma-Aldrich), and 0.2 mM ATP). The time of the addition of TIRF buffer or FL-HTT to the actin mix was defined as *t* = 0. Samples were observed on an inverted microscope (Eclipse Ti, Nikon) equipped with an Ilas2 TIRF system (Roper Scientific), a cooled charge-coupled device camera (EMCCD Evolve 512, Photometrics), and a warm stage controller (LINKAM MC60) and controlled by MetaMorph software (version 7.7.5, Molecular Devices). Samples were excited with 565-nm laser light, and time-lapse imaging (at 590 nm) was performed at 26°C for actin polymerization, during 30 min at 1 frame per 10 s with a 100-ms exposure time.

### STORM imaging for in vitro bundling

The F-actin prepolymerized in a previously described way was first incubated with 125 nM FL-HTT, HTT_N-B, and HTT_B-C fragments and the Basic1mut. Subsequently, the mixture was attached to a PLL-precoated glass-bottom dish for 18 min at RT. After being washed twice with Dulbecco’s phosphate-buffered saline (DPBS), the attached sample was fixed using 3% (v/v) paraformaldehyde (PFA; Electron Microscopy Sciences) and 0.1% (v/v) glutaraldehyde (Electron Microscopy Sciences) in DPBS for 10 min at RT. To remove the unreacted aldehyde groups, the fixed sample was washed with DPBS and then treated with freshly prepared 0.1% (w/v) NaBH_4_ Sigma-Aldrich) for 7 min at RT. After a brief washing with DPBS, the sample was incubated in a 2.5 μM Silicon Rhodamine (SiR)-actin dye solution (Cytoskeleton) in DPBS for 1 hour at RT.

For STORM imaging, SiR-actin–labeled actin filament samples were immersed in DPBS containing 100 mM cysteamine (Sigma-Aldrich), 5% (w/v) glucose, and oxygen-scavenging enzymes [glucose oxidase (0.5 mg/ml; Sigma-Aldrich) and catalase (38 g/ml; Sigma-Aldrich)] at pH 8.5. STORM imaging was performed using a custom-built inverted microscope (Ti2-U; Nikon) with a 100× 1.49 numerical aperture (NA) oil immersion objective lens (CFI SR HP Apo TIRF; Nikon). A 647-nm laser (120 mW, OBIS; Coherent) was used to continuously illuminate the prepared sample to excite the SiR dye molecules. The sample was excited using TIRF illumination, and the fluorescence emission from the sample was filtered using a bandpass emission filter (LF 408/488/561/635-B; Semrock). An EMCCD camera (iXon Ultra 888; Andor) was used to detect the filtered fluorescence at a frame rate of 65 Hz. We used a CRISP Autofocus System (Applied Scientific Instrumentation, ASI) to maintain the focal plane during STORM imaging. After collecting a raw STORM video, each point spread function shown in a raw image was fitted to a Gaussian function to determine the centroid positions of each fluorophore, allowing for the reconstruction of the STORM image. The collected centroid positions of each molecule were drift-corrected and then rendered for the final STORM image. For width measurements of individual actin filaments, the cross-sectional profile of each filament was generated from STORM images, and then the full width at half maximum was measured as the width of the actin filaments.

### Cell culture

#### Neuronal primary culture

Cortices were dissected from embryonic day 15.5 (E15.5) to E16.5 embryos and digested in papain enzyme solution. Papain was inactivated using 10% fetal bovine serum (FBS), and cells were washed with opti-MEM glucose. Cells were cultured on poly-l-lysine matrices in neurobasal supplemented with 2% B27, 1% GlutaMAX, 1% penicillin/streptomycin, and 10% FBS for 1 hour, and medium was replaced by neurobasal containing 2% B27, 1% GlutaMAX, and 1% pennicillin/streptomycin. Neurons were incubated in 5% CO_2_ in a humidified incubator at 37°C. For Western blot analysis, cells were plated at 2,000,000 cells in a P10 petri dish and were lysed at indicated DIV.

For analysis of endogenous HTT expression and localization, we used embryos from the Swiss/CD1 mouse strain (Janvier Lab). For permeabilization assays, Swiss/CD1 neurons were electroporated/nucleofected at DIV0 with 3 μg of plasmid encoding for mVENUS (pCAG-mVENUS pSCV2, provided by J. Courchet, Neuromyogene, Lyon, France) and mixed 1:1 with nonelectroporated cells to favor cell survival. To assess the effects of HTT depletion, Hdh^lox/lox^ mouse (*55*) embryos were electroporated at DIV0 just before plating with 3 μg of the plasmid control encoding for Td-tomato alone (pCAG td Tomato, Addgene) or 3 μg of plasmid encoding for Cre recombinase and reporter gene Td-tomato (pCAG td Tomato-Ires-Cre, Gage’s laboratory). To assess HTT down-regulation by immunoblotting and analyze growth cone morphology, electroporated cells were mixed (1:1) with nonelectroporated cells. For axonal length and branching analysis, electroporated cells were mixed (1:4) with nonelectroporated cells. For immunofluorescence analysis, cells were plated at 800,000 cells per well of a six-well plate and were fixed at DIV4. For the axonal growth rescue experiment, HTT-depleted neurons were infected at DIV1 with lentiviral constructs expressing HTT_C or HTT_NB domain. Neurons were fixed at DIV4 and analyzed with 20× objective on a Nanozoomer S60 slide scanner.

#### Mouse embryonic fibroblast

MEFs were obtained following the protocol in (*56*). Briefly, fibroblasts were dissected from the forelimb and hindlimb buds of E14.5 embryos and digested in a 0.25% trypsin enzyme solution. Trypsin was inactivated using 10% FBS–Dulbecco’s modified Eagle medium (DMEM). The cells were cultured in high-glucose DMEM supplemented with 10% FBS and 1× penicillin-streptomycin. After isolation, the MEFs were passaged every 2 to 3 days, and experiments were conducted 4 to 6 days postisolation. The fibroblasts were incubated in a humidified incubator at 37°C with 5% CO_2_.

#### COS-7 cells

COS-7 cells were cultured in DMEM supplemented with 10% FBS. Twenty-four hours before transfection, cells were seeded in six-well plates containing glass coverslips at 70% confluence. COS-7 cells were transfected with 1 μg of plasmid using Lipofectamine 3000 according to the manufacturer’s instructions. After 24 hours, the cells were fixed with 4% PFA and 4% sucrose in cytoskeleton buffer prewarmed to 37°C.

### Immunofluorescence

Cells were fixed at DIV4 with 0.5% glutaraldehyde (Sigma-Aldrich), 0.1% Triton X-100 (Sigma-Aldrich) in cytoskeleton buffer [10 mM MES, 138 mM KCl, 3 mM MgCl_2_, and 2 mM EGTA (pH 6.1)] supplemented with sucrose (10%) and prewarmed at 37°C. Glutaraldehyde autofluorescence was quenched by 10-min incubation in sodium borohydride (1 mg/ml). Blocking was performed with PBS–5% BSA for 1 hour at RT. Primary antibodies were incubated overnight at 4°C. Secondary fluorochrome-conjugated antibodies were applied for 1.5 hours at RT and phalloidin (1:1000) for 30 min at RT. Glutaraldehyde autofluorescence was quenched by 10-min incubation with PBS supplemented with sodium borohydride (Sigma-Aldrich). Washes were done in PBS, and coverslips were mounted in Dako fluorescent mounting medium (Agilent). The following antibodies and reagents were used: mouse monoclonal anti-HTT (4C8, Institut de Génétique et de Biologie Moléculaire, IGBMC, Strasbourg), diluted 1:100; rat monoclonal anti-tyrosinated tubulin antibody (YL1/2, provided by I. Arnal, Grenoble Institute Neuroscience), diluted 1:4000; chicken anti-GFP (Abcam), diluted 1:1000; phalloidin-ATTO488 or phalloidin-ATTO647 (Sigma-Aldrich) to stain F-actin, diluted 1:1000 and chicken anti-GFP (Abcam) diluted 1:1000. Secondary antibodies were donkey anti-mouse immunoglobulin G (IgG; H + L)–Cy3 conjugate (Jackson ImmunoResearch), donkey anti-rat IgG (H + L)–Cy5 conjugate (Jackson ImmunoResearch), and chicken IgY (H + L)–Alexa Fluor 488 (Thermo Fisher Scientific).

### Confocal imaging

Growth cones were visualized with a confocal laser scanning microscope (Zeiss, LSM 710) and a GaAsP detector (Zeiss Airyscan) using a ×63 oil-immersion objective (NA, 1.4). The confocal stacks were deconvolved with AutoDeblur. To measure axonal length, we took large-field images using an ×20 objective (Zeiss, Axioscan or mosaic mode on Zeiss Confocal LSM710). Acquisitions were performed in the photo-imaging facility of the Grenoble Institute of Neuroscience.

### Morphologic/morphometric analyses

To assess axon length, we defined the axon as the neuron’s longest neurite, which had to measure at least 100 μm. (Lengths were measured using NeuronJ plugin in ImageJ software.) For branching analysis, we counted manually the number of axon primary branches that were at least 15 μm long and normalized them to the length of the axon bearing them. Only neurons having at least one branch were counted. For growth cone morphology analyses, filopodia were defined as actin filaments emerging at least 1 μm beyond the lamellipodial structure, which delimited the actin crown perimeter. The growth cone area was measured from the axonal shaft to the end of the area delimitated by phalloidin staining. Exploratory microtubules were measured from their tips to the main microtubule bundle in the central domain.

### Permeabilization assay

At DIV4, cells were treated at 37°C for 3 min with 10 μM paclitaxel/taxol (Sigma-Aldrich) in BRB80 buffer that was supplemented or not with 0.05% Triton X-100 (Sigma-Aldrich) for the permeabilized or control conditions, respectively. After treatment, cells were immediately fixed as described above. Growth cones were immunostained for endogenous HTT and F-actin and imaged as described above, with the same parameters and the mean fluorescence intensities of Venus, HTT, and phalloidin signals measured within the growth cone.

### Quantification and statistical analysis

GraphPad Prism (GraphPad Software Inc.) software was used for statistical analyses. Outliers were identified by using ROUT method with *Q* = 1% and removed for following statistical analysis. Normality of data distribution was assessed on control conditions by applying the d’Agostino-Pearson omnibus normality test. The parametric *t* test was applied when data followed a normal distribution; the nonparametric *t* test was used for non-normal data distribution. Paired or unpaired *t* tests were applied. One-way analysis of variance (ANOVA) multiple comparisons was applied for recombinant protein actin binding and bundling assay analysis.
