# Supplementary material for: Structure of the Huntingtin F-actin complex reveals its role in cytoskeleton organization
Source: Sci Adv. 2025 Sep 19;11(38):eadw4124. doi: 10.1126/sciadv.adw4124 (PMC12448071; doi:10.1126/sciadv.adw4124)
Supplement: Supplementary file 1 — Figs. S1 to S11 Table S1 Legends for movies S1 and S2 [file sciadv.adw4124_sm.pdf]

Science Advances
AAAS

Supplementary Materials for

Structure of the Huntingtin F-actin complex reveals its role in cytoskeleton organization

Rémi Carpentier *et al.*

Corresponding author: Mariacristina Capizzi, m.capizzi@icm-institute.org; Florian K. M. Schur, florian.schur@ist.ac.at; Ji-Joon Song, songj@kaist.ac.kr; Sandrine Humbert, sandrine.humbert@icm-institute.org

Sci. Adv. **11**, eadw4124 (2025)
DOI: 10.1126/sciadv.adw4124

The PDF file includes:

Figs. S1 to S11
Table S1
Legends for movies S1 and S2

Other Supplementary Material for this manuscript includes the following:

Movies S1 and S2

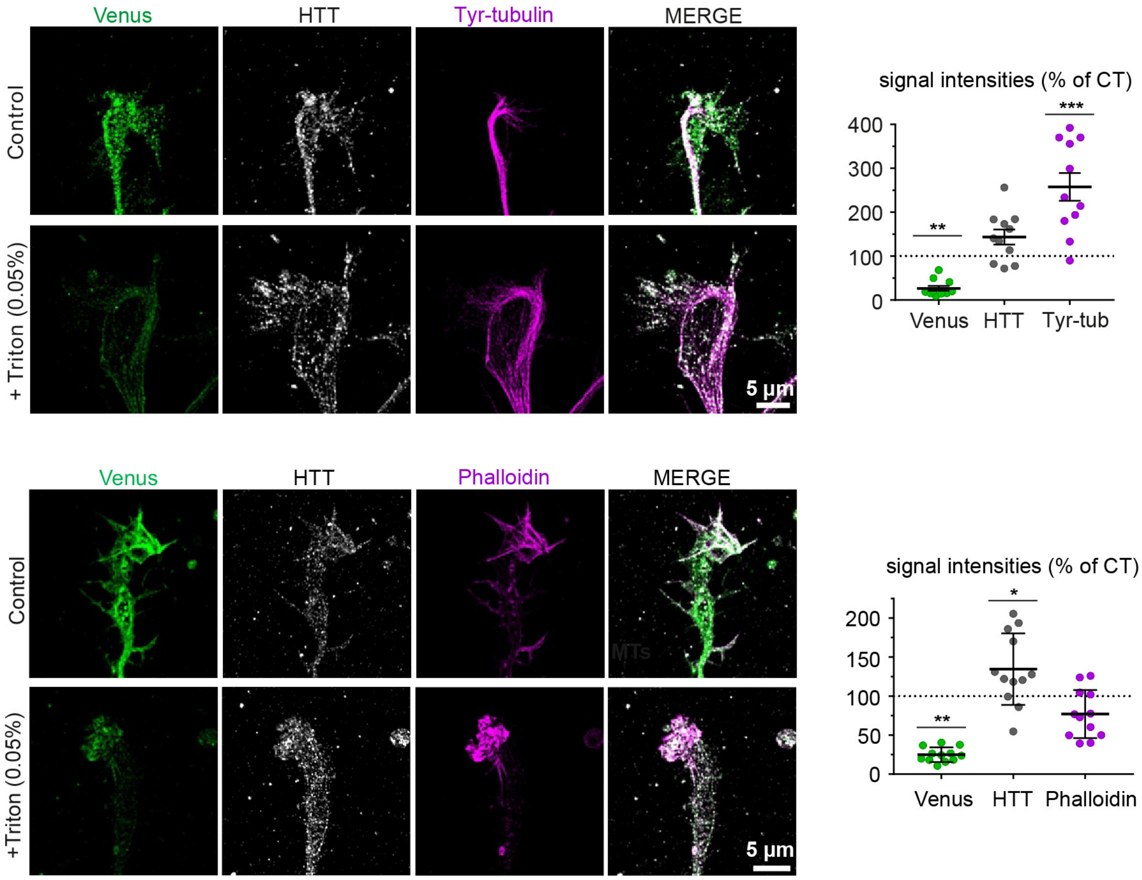

Fig. S1. HTT associates with cytoskeleton in growth cones. *Top:* Representative confocal images of growth cones of control neurons (CT, no Triton X100) and permeabilized (+Triton X100) Venus-positive neurons (green) immunostained for endogenous HTT (grey) and microtubules (Tyrosinated tubulin, magenta). Scale bar, 5 µm. Quantification of mean fluorescence intensity of indicated signals within growth cones after permeabilization. Unpaired t-test, **p <0.01; ***p <0.001 (Venus: 26.61 ±8.36; HTT: 143.50 ±56.04; Tyr-tub: 257.50 ±105.30; n=11 growth cones/condition). *Bottom:* Representative confocal images of growth cones of control neurons (CT, no Triton X100) and permeabilized (+Triton X100) Venus-positive neurons (green) immunostained for endogenous HTT (grey) and F-actin (phalloidin, magenta). Scale bar, 5 µm. *Right:* Mean fluorescence intensity ±SD of indicated signals within growth cones after permeabilization. Unpaired t-test, *p <0.05, **p <0.01 (Venus: 24.88 ±9.33; HTT: 134.50 ±45.80; Phalloidin: 76.88 ±30.93; n=12 growth cones/condition).

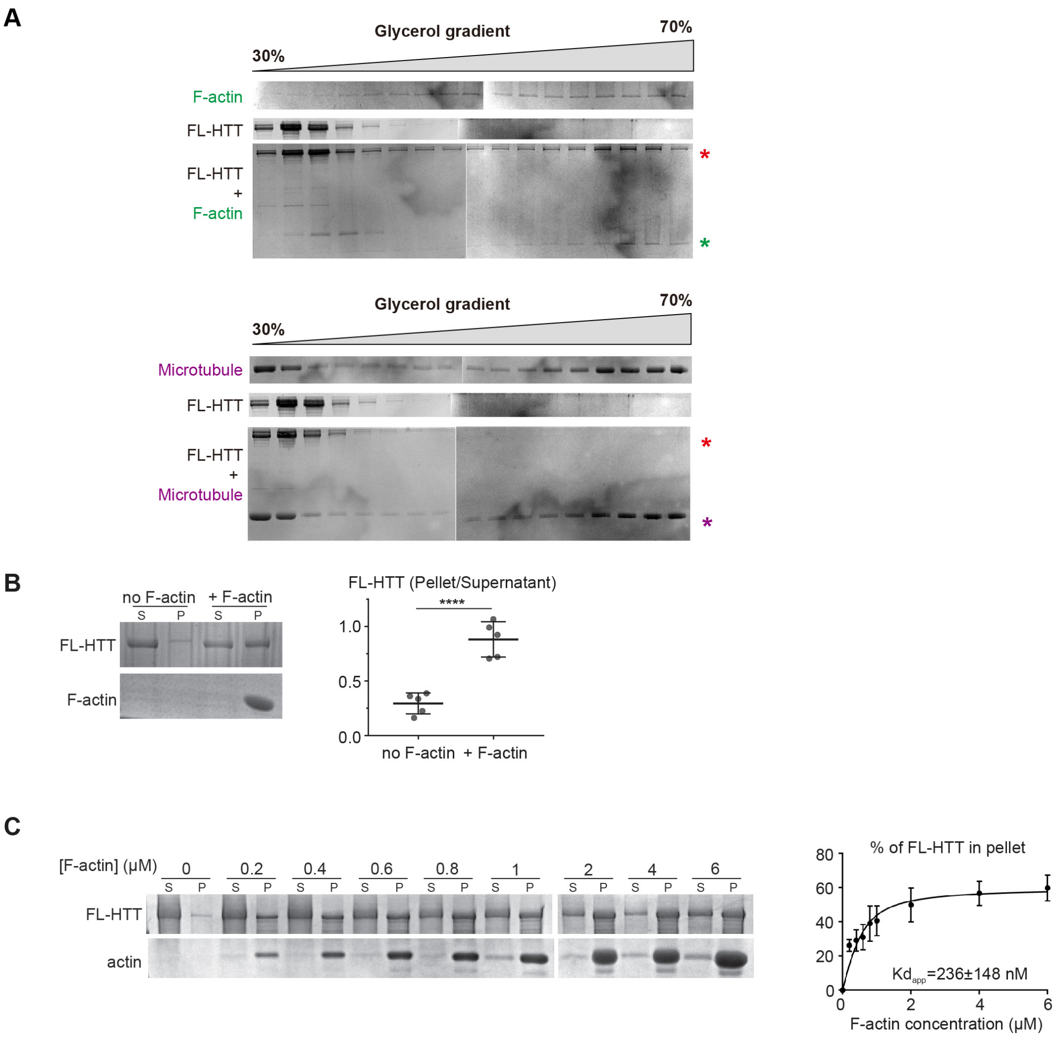

Fig. S2. HTT directly binds to F-actin. (A) A representative SDS-PAGE of fractions obtained after ultracentrifugation on continuous glycerol gradient of the FL-HTT incubated with or without F-actin (top) and microtubule (bottom). F-actin, HTT and microtubule bands are marked by green, red and purple asterisks, respectively. (**B**) *Left:* Representative SDS-PAGE stained with Coomassie blue. S, supernatant; P, pellet. *Right:* Graph shows the amount of FL-HTT found in the pellet with or without F-actin. Paired t-test, **** $p < 0.0001$(No F-actin: 0.29 ±0.10; + F-actin: 0.88 ±0.16; from 5 independent experiments). (**C**) Determination of FL-HTT binding affinity for F-actin. *Left:* Representative SDS-PAGE of FL-HTT incubated with an increasing amount of F- actin. *Right:* Graph shows the bound fraction of FL-HTT expressed as the percent of total FL-HTT, Pellet+Supernatant relative to F-actin concentration.

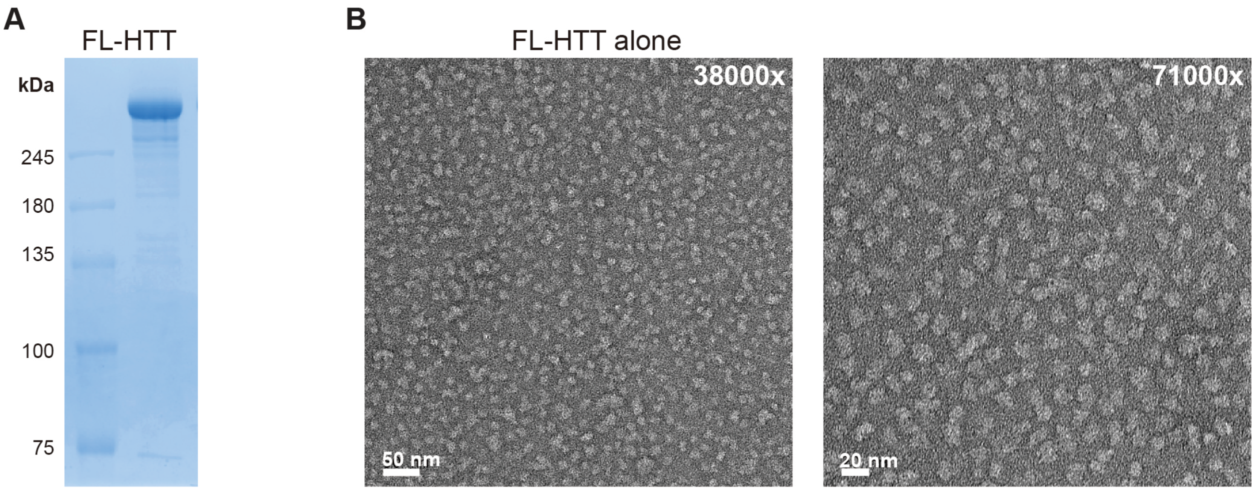

Fig. S3. Purified FL-HTT and a representative Negative stain EM image of FL-HTT. (**A**) Purified FL-HTT shown in SDS-PAGE. (**B**) a representative Negative stain EM image of FL-HTT.

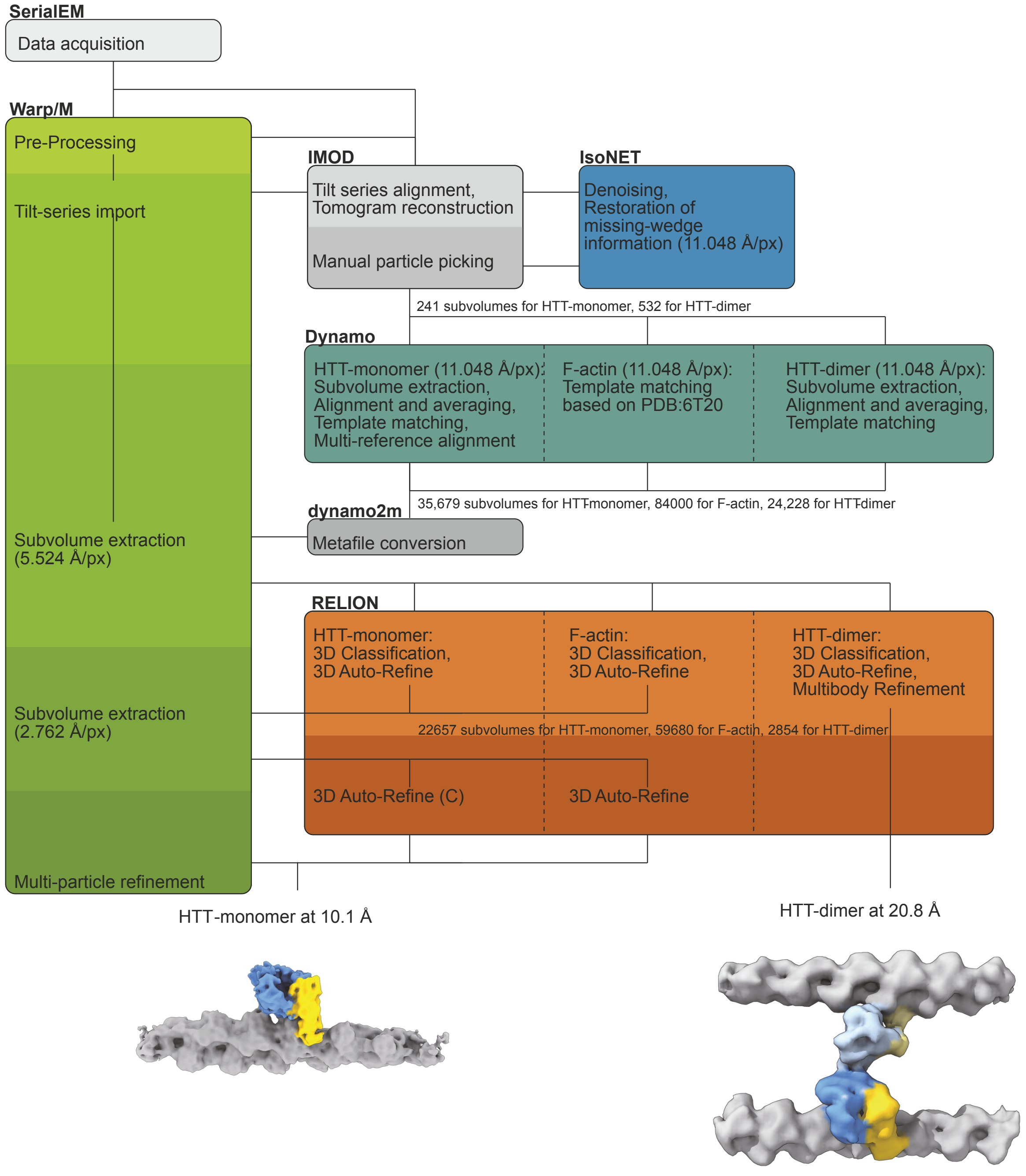

Fig. S4. The scheme for cryo-ET and STA processing.

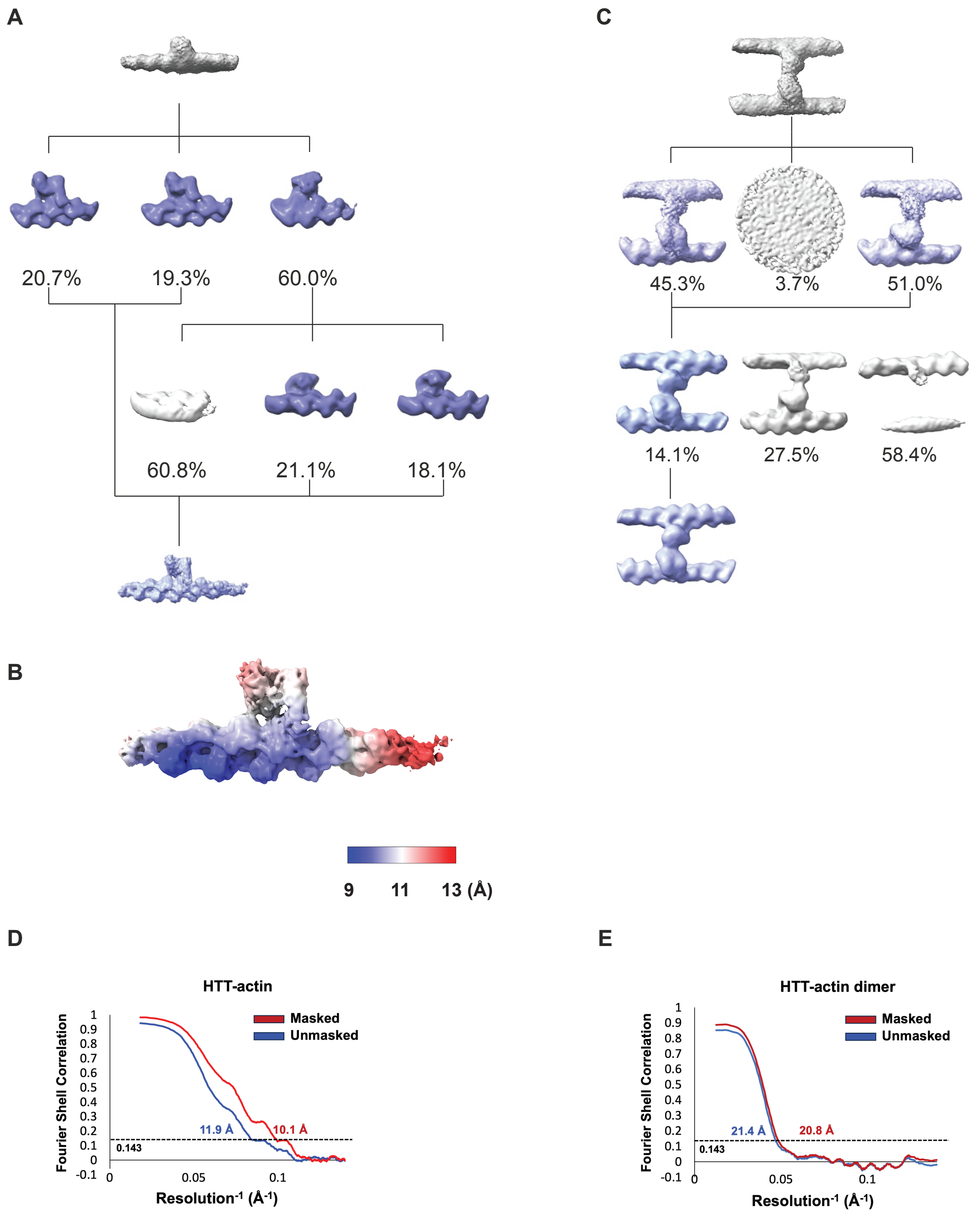

Fig. S5. Cryo-EM data processing workflow. (**A**) 3D classification and refinement of HTT/F-actin complex. (**B**) The final cryo-EM map colored according to the local resolution. (**C**) 3D classification and refinement of HTT dimer/F-actin complex. (**D, E**) The gold standard FSC (criteria at 0.143) indicate 10.1 and 20.8 Å resolutions for HTT/F-actin and HTT/F-actin dimer complexes, respectively.

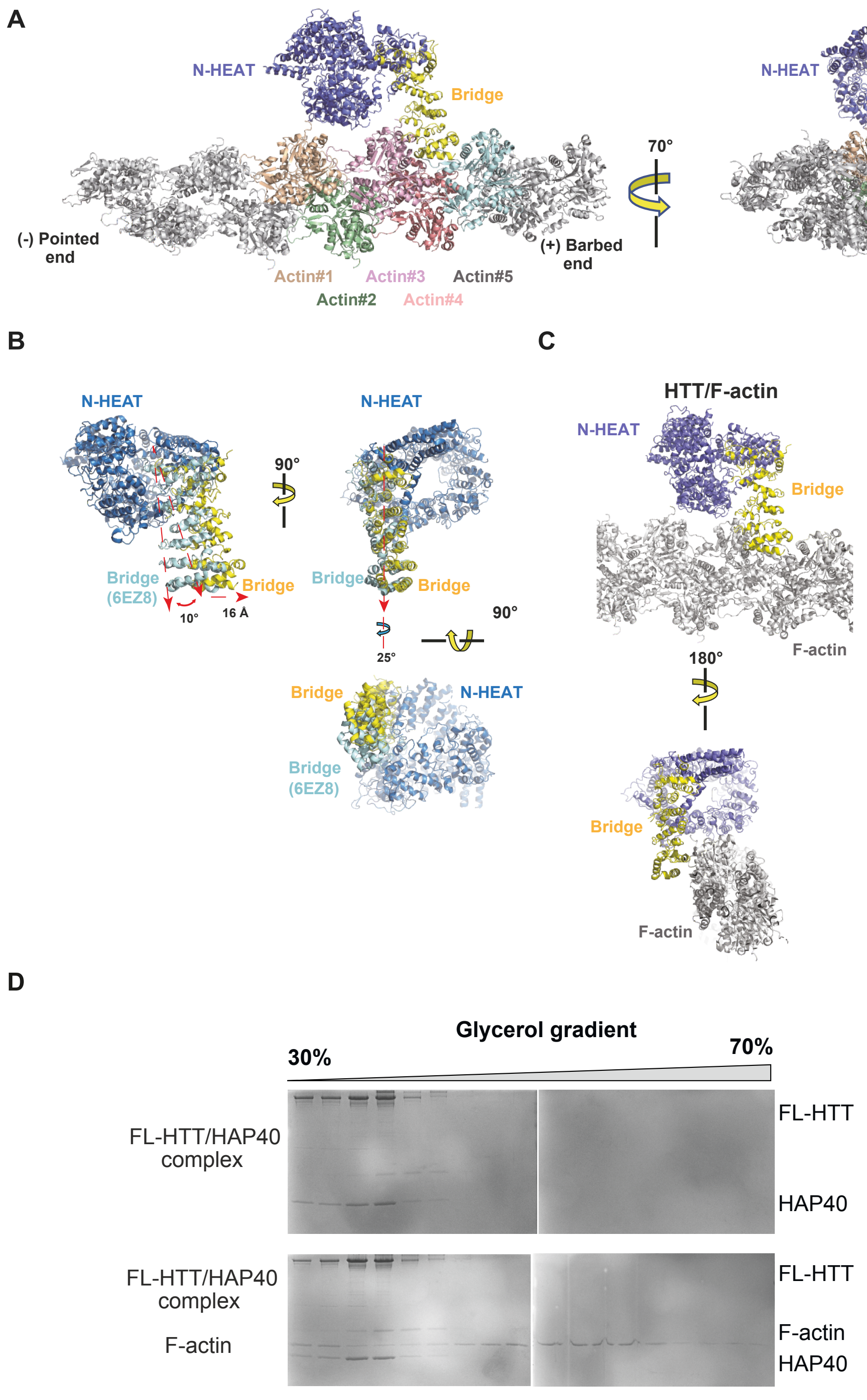

Fig. S6. HTT undergo conformational change upon F-actin binding. (**A**) Molecular model of HTT bound to F-actin. (**B**) Conformational change of the Bridge-domain compared to the HTT-HAP40 model upon F-actin binding. (**C**) Comparison of the binding modes of HTT-F-actin complex and HTT-HAP40 complex. (**D**) A representative SDS-PAGE of fractions obtained after ultracentrifugation on glycerol gradient of FL- HTT/HAP40 complex incubated with or without F-actin. FL-HTT/HAP40 does not bind to F-actin.

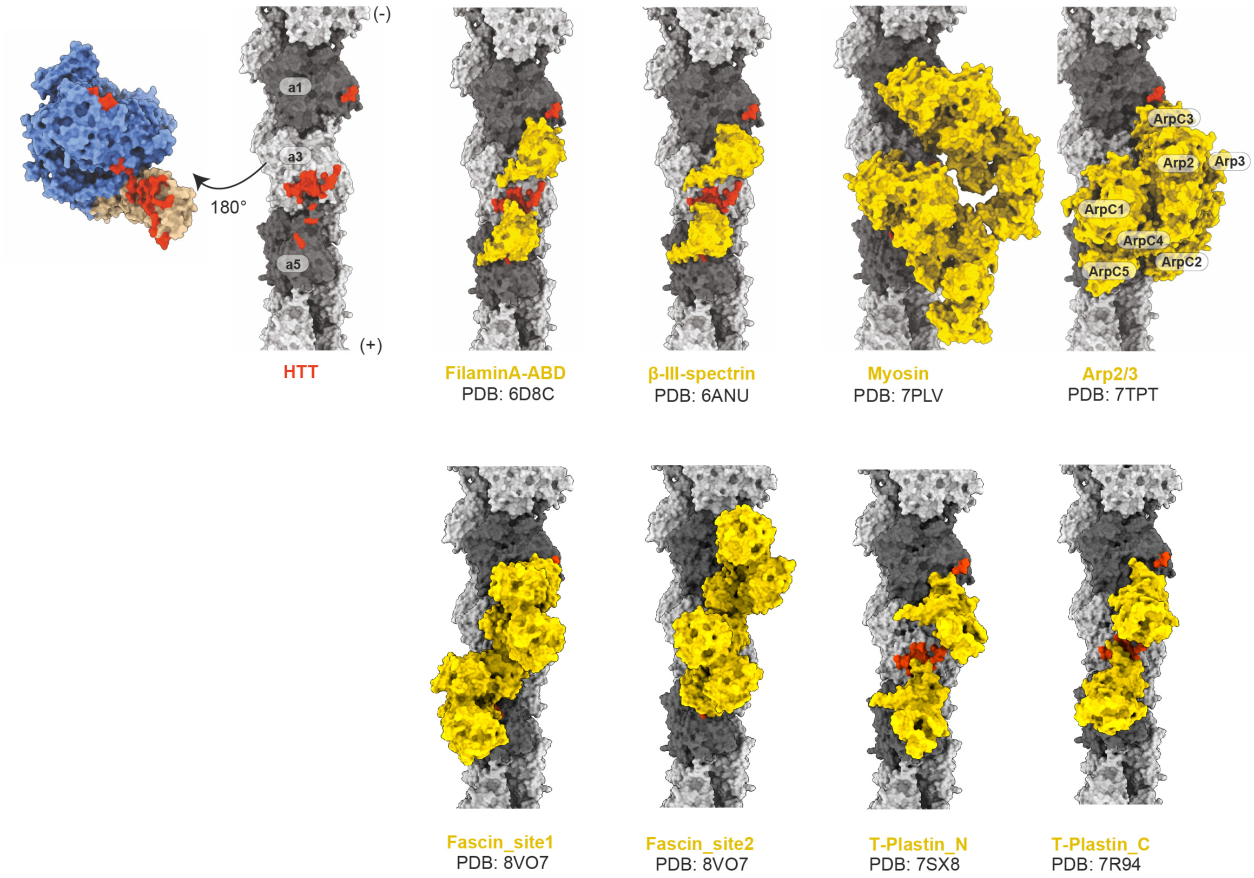

Fig. S7. Comparison of the F-actin interaction mode of HTT and other ABPs. Structural gallery comparing the surface covered by HTT on F-actin with those interfaces covered by other known ABPs. The direct interaction interface between HTT and F-actin is colored in red; other ABPs are shown as surface models in yellow.

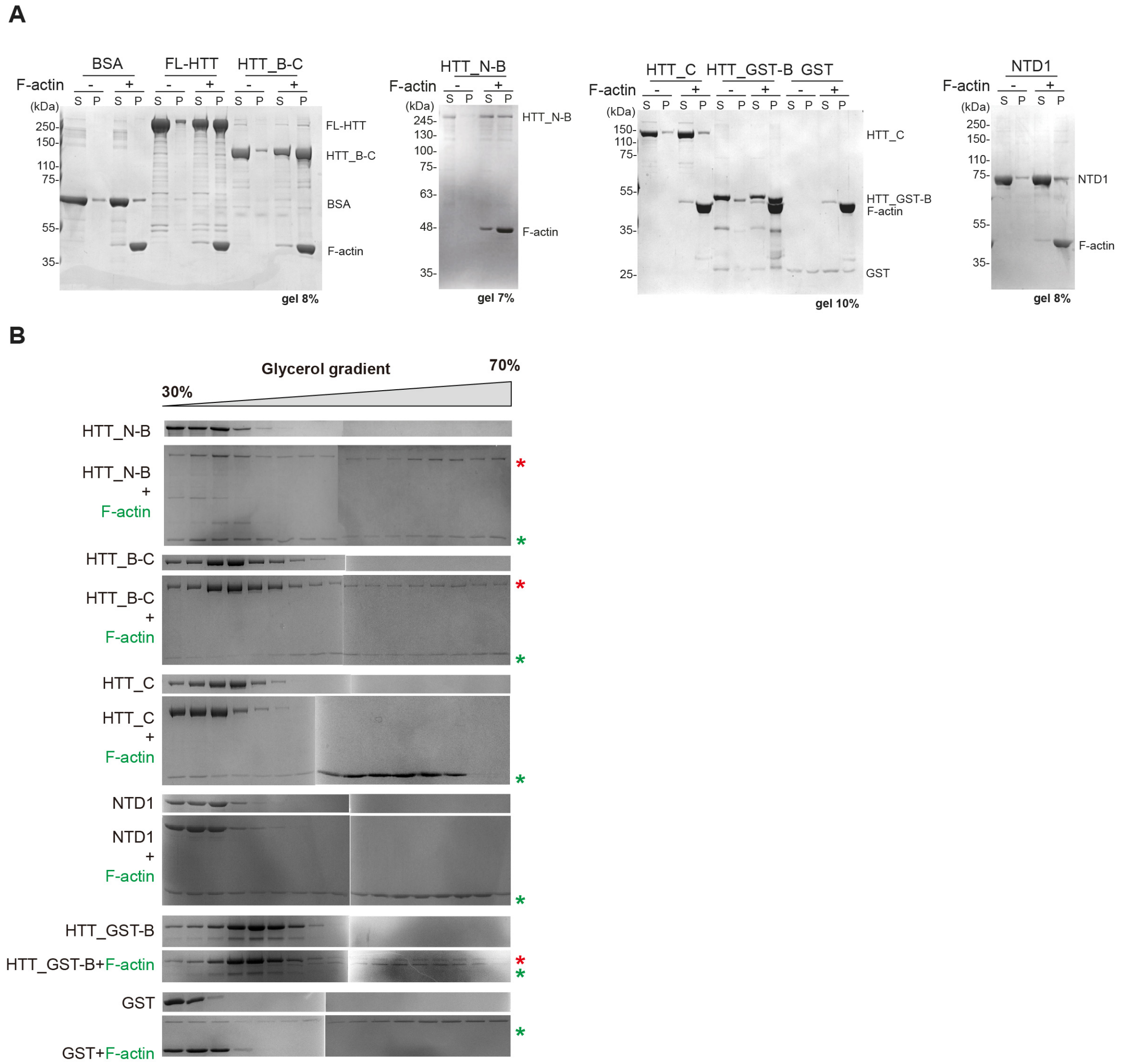

Fig. S8. The interaction between HTT and F-actin. (**A**) *Top row:* Representative Coomassie blue-stained SDS-PAGE of high-speed co-sedimentation assay with different HTT fragments and F-actin. S, supernatant; P, pellet. (**B**) A representative SDS-PAGE of fractions obtained after ultracentrifugation on continuous glycerol gradient of the different HTT fragments incubated with or without F-actin. The actin bands are marked by green asterisks and the HTT by red asterisks.

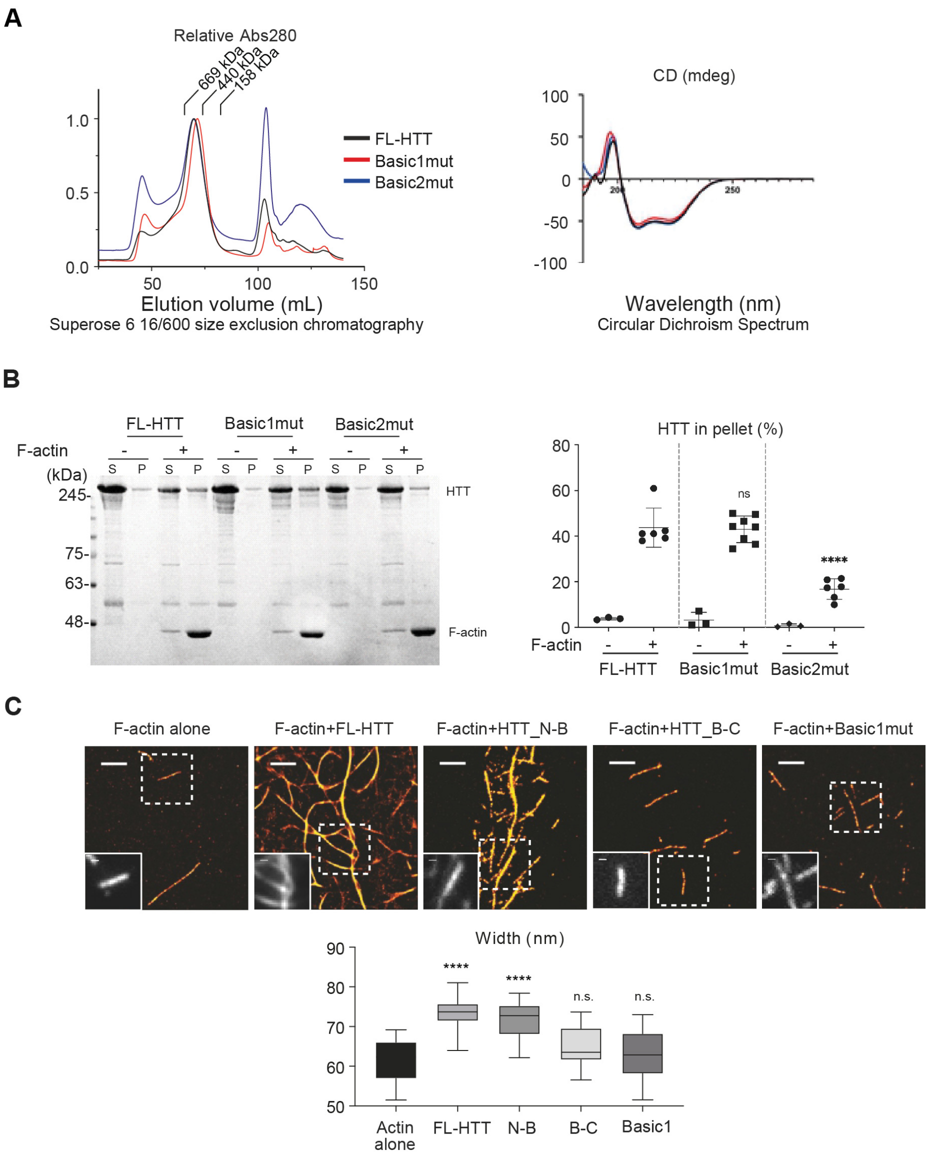

Fig. S9. Mutation analysis of HTT and F-actin interactions. (**A**) Gel-filtration and circular dichroism profiles of FL-HTT, Basic1mut and Basic2mut. (**B**) *Left:* Representative SDS-PAGE image of high-speed co-sedimentation assay with FL-HTT, Basic1mut, and Basic2mut with or without F-actin. S, supernatant; P, pellet. *Right*: Quantification of FL-HTT, Basic1mut, and Basic2mut in the pellet in the presence of F-actin. Graph shows the percent of HTT in the pellet. One-way ANOVA test followed by Dunnet's post hoc test, ****$p<0.0001$ ($n>=3$). Data are shown as mean ±SD. (**C**) Super-resolution fluorescence imaging of F-actin stained with silicon rhodamine dye-labeled jasplakinolide (SiR-actin) alone or in presence of 125 nM of FL-HTT, HTT_N-B, HTT_B-C, or Basic1mut, using stochastic optical reconstruction microscopy (STORM) at a time point of 18 min. *Inset*: diffraction-limited fluorescence images of the white dashed boxes. Scale bars, 2 µm and 500 nm (inset). *Bottom:* Graph shows STORM image analysis of F-actin object width in the presence of FL-HTT, HTT_N-B, HTT_B-C and Basic1mut.Non-parametric Kruskal-Wallis test, **** $p<0.0001$, paired t-test (Actin alone: 62.32 nm: n=48, FL-HTT: 73.68 nm; n=32, HTT_N-B: 72.73 nm; n=45, HTT_B-C: 63.50 nm; n=37, Basic1mut: 62.86 nm; n=37). Data are shown as box-whisker plots.

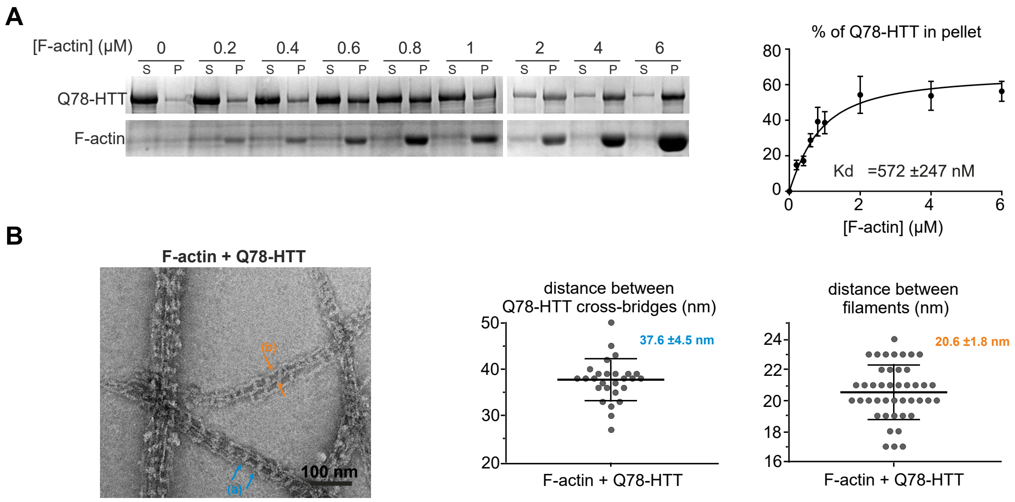

Fig. S10. Polyglutamine-expanded Q78 HTT binds and bundles F-actin. (**A**) Determination of Q78-HTT binding affinity for F-actin. *Left:* Representative SDS-PAGE of Q78-HTT incubated with increasing amounts of F-actin. *Right:* Graph shows the bound fraction of Q78-HTT expressed as the percent of total Q78-HTT (Pellet+Supernatant) relative to F-actin concentration (0 µM: 0.0 ±0.0%; 0.2 µM: 16.88 ±4.15%; 0.4 µM: 18.06 ±6.20%; 0.6 µM: 32.25 ±2.85%; 0.8 µM: 46.40 ±9.05%; 1 µM: 44.15 ±6.96%; 2 µM: 61.73 ±17.90%; 4 µM: 52.01 ±9.34% ; 6 µM: 55.24 ±12.76%). Kd_{app}, calculated dissociation constant at equilibrium from 3 independent experiments. (**B**) *Left:* A representative electron micrograph of negative staining showing F-actin bundles in the presence of Q78-HTT: (a) shows the distance between two HTT cross-bridges and (b) between two actin filaments within a bundle. *Right:* Graphs show the spacing between Q78-HTT cross-bridges (37.59 ±4.53 nm; from 3 independent experiments), the distance between actin filaments within a bundle (20.58 ±1.76 nm; from 3 independent experiments).

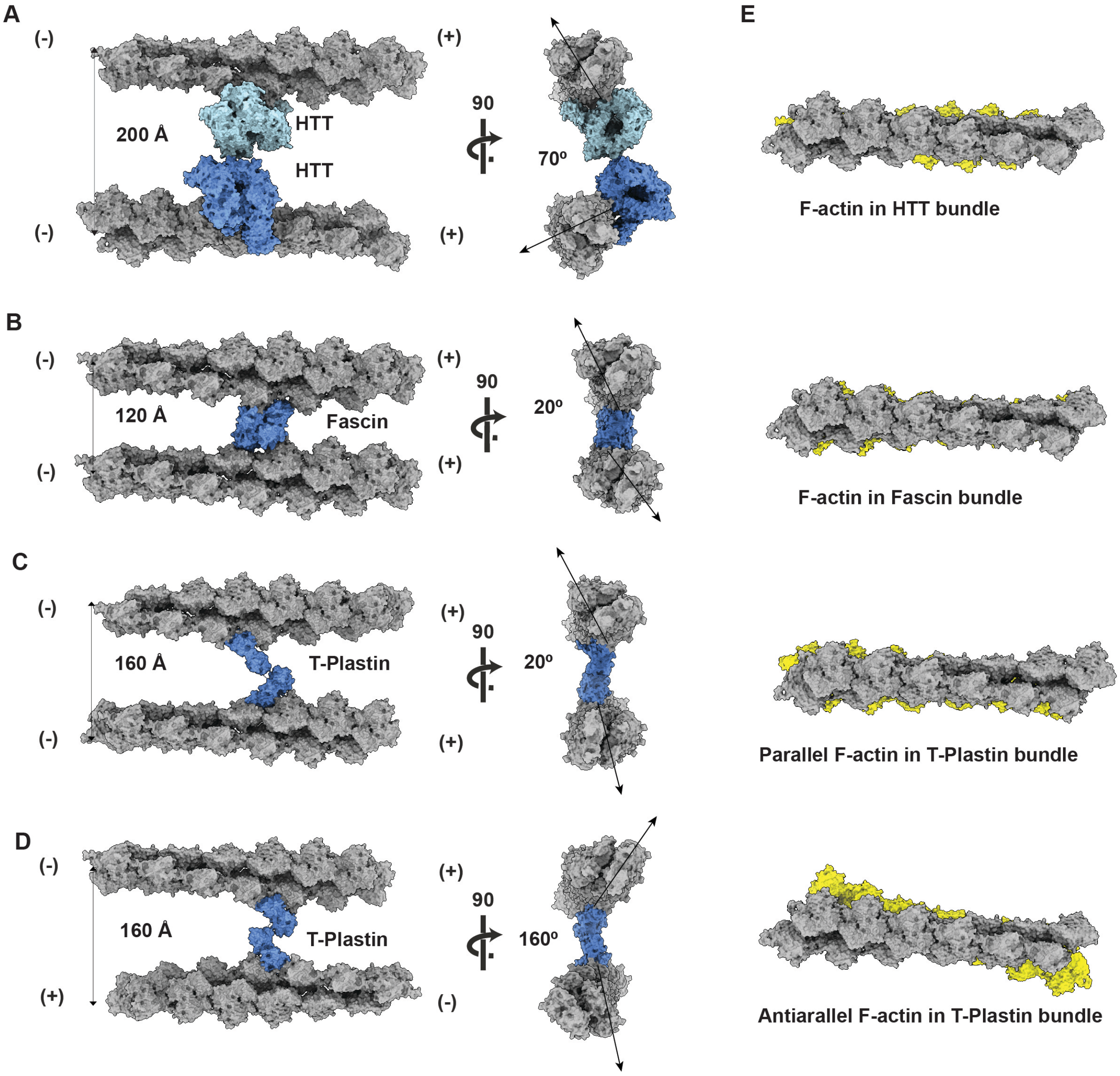

Fig. S11. Comparative analysis of actin bundles mediated by HTT, Fascin and T- Plastin.
The atomic models of the cryo-EM/ET structure of HTT_F-actin (from this study) (**A**), Fascin_F-actin (PDB ID: 8VO7) (**B**), and T-Plastin_F-actin in parallel (PDB ID: 7SX8) (**C**) and anti-parallel (PDB ID: 7SX8) (**D**) configurations. The distances of two actin filaments are indicated with the orientation of F-actin (-: pointed, +: barbed ends) in the left panel. The right panels show that the relative orientation and angles of two actin-filaments. (**E**) The views for the two actin-filaments aligned show the degree of skewness of the bundles.

Table S1. Parameters of structural data acquisition and image processing.

**Sample**	Q21 HTT-Actin complex	
**Data collection**		
Microscope	Thermo Fisher Titan Krios G3i TEM	
Voltage (keV)	300	
Detector	Gatan BioQuantum K3	
Energy-filter slit width (eV)	20	
Magnification (nominal)	64,000x	
Pixel size (Å/pixel)	1.381	
Defocus range (μm)	-2 to -6	
Defocus step (μm)	0.5	
Acquisition scheme	-/+ 60, 2 step (dose-symmetric)	
Total Dose (e-/Å^2)	170	
Frame number	8	
Number of Tomograms used	42	
**Data processing**		
	**HTT-actin monomer** (EMD-39097, PDB 8YAE)	**HTT-actin dimer** (EMD-39103, PDB 8YAO)
Initial subvolumes from template matching	84,019	21,000
Final subvolumes	22,664	2,854
Symmetry imposed	C1	C1
Final resolution (0.143)	10.10 Å	20.80Å
Map sharpening B factor (Å^2)	-5.47	-5.47
Initial model used	Huntingtin (6EZ8), Actin (7NEP)	
Refinement package	Phenix	
**Model-map scores**		
CC (Calculated in ChimeraX)	0.85	0.91
**Model composition**		
Non-hydrogen atoms	49,440	98,880
Protein residues	6,313	12,626
R.m.s. deviations		
- Bond lengths (Å)	0.015	0.015
- Bond angles (°)	2.156	2.156
C-beta outliers (%)	0.97	0.96
**Model Validation**		
MolProbity score	1.84	2.00
Clashscore	5.85	9.04
Poor rotamers (%)	1.57	1.57
**Ramachandran Plot**		
Favored	94.59	94.59
Allowed	4.36	4.36
Outliers	1.05	1.05

Movie S1. TIRF time-lapse movies of ATTO565-actin polymerization.

Representative TIRF time-lapse movies of ATTO565-actin polymerization with 1.5 μM of actin alone (left), and 1.5 μM of actin and 125 (middle) or 500 nM (right) of FL-HTT. Frames were taken at 10 sec intervals over periods of 30 min (see frame images in Fig. 2C). Scale bar, 20 μm.

Movie S2. Molecular model of the HTT F-actin complex.

F-actin is in gray, the N-HEAT and Bridge domains are in blue and yellow, respectively.
